# Synthesis and Characterization of Oligothiophene–Porphyrin-Based Molecules That Can Be Utilized for Optical Assignment of Aggregated Amyloid-β Morphotypes

**DOI:** 10.3389/fchem.2018.00391

**Published:** 2018-09-03

**Authors:** Katriann Arja, Mathias Elgland, K. Peter R. Nilsson

**Affiliations:** Division of Chemistry, Department of Physics, Chemistry and Biology, Linköping University, Linköping, Sweden

**Keywords:** oligothiophene, porphyrin, protein deposits, imaging, fluorescence

## Abstract

Molecular tools for fluorescent imaging of protein aggregates are essential for understanding the significance of these pathological hallmarks in proteopathic neurodegenerative diseases, such as Alzheimer's disease. Here, we report the synthesis of a series of oligothiophene porphyrin hybrids, OTPHs, and the evaluation of these dyes for fluorescent imaging of beta-amyloid aggregates in tissue sections from a transgenic mouse model with Alzheimer's disease pathology. The OTPHs proved to be successful for spectral and lifetime imaging assessment of protein deposits and our findings confirm that the enhanced spectral range and distinct lifetime diversity of these novel tools allow a more precise assessment of heterogeneous amyloid morphology compared with the corresponding oligothiophene dye. In addition, the chemical identity of the porphyrin moiety, as well as the spacing between the two optical active moieties, influenced the OTPHs performance for fluorescent assignment of the protein deposits. We foresee that our findings will aid in the chemical design of dyes that can be utilized as optical tools for studying the polymorphic nature of protein aggregates associated with proteopathic neurodegenerative diseases.

## Introduction

Fluorescent molecules for selective detection of protein aggregates are of great interest as such protein deposits are associated with devastating and progressive neurodegenerative diseases, such as Alzheimer's disease (AD) (Ross and Poirier, 2004; Chiti and Dobson, 2006). The two characteristic histopathological hallmarks of AD are amyloid-β (Aβ) plaques and neurofibrillary tangles (NFTs) composed of hyperfosforylated tau protein (Ballatore et al., 2007). The ability to detect and assign these protein deposits gives the opportunity to study the biochemical processes and structural changes involved in the aggregate formation, as well as the underlying molecular mechanisms of the pathogenesis with the possibility to achieve better diagnostics and potential treatment toward AD. In this regard, the development of fluorescent dyes for optical imaging of disease-associated protein aggregates is essential.

A plethora of small hydrophobic fluorescent molecules targeting disease-associated proteinaceous deposits has been reported (Klunk et al., 1995; Styren et al., 2000; Kung et al., 2001; Mathis et al., 2004; Nesterov et al., 2005; Furumoto et al., 2007; Ono et al., 2012). Lately, luminescent conjugated oligothiophenes (LCOs) consisting of repetitive monomeric thiophene units, varying in length and in side chain functionalities, have also been presented and these dyes can be utilized for detecting and studying protein aggregates both *in vitro* and *in vivo* (Åslund et al., 2009; Klingstedt et al., 2011, 2013; Simon et al., 2014; Shirani et al., 2015; Bäck et al., 2016). This class of molecules has been shown to identify a greater diversity of disease-associated protein aggregates compared to conventional dyes. Furthermore, due to the conformation sensitive intrinsic fluorescent properties of the LCOs, spectral assignment of distinct aggregated morphotypes can also been achieved. Thus, the LCO specific optical signatures can be used not only for detection of protein deposits but also for distinguishing between different types of protein deposits and for visualizing structural heterogeneity within the same protein deposit (Wegenast-Braun et al., 2012; Klingstedt et al., 2015; Rasmussen et al., 2017). Molecular tool for assigning multiple protein aggregate morphotypes might be highly important since recent studies have revealed inter-subject variability of Aβ aggregates in familial and sporadic AD, as well as that different aggregate species of Aβ are present during different stages of the pathological process (Nyström et al., 2013; Cohen et al., 2015; Klingstedt et al., 2015; Rasmussen et al., 2017; Condello et al., 2018). In addition, Aβ aggregates extracted from two AD patients with distinct clinical history and pathology displayed two different structures, suggesting a correlation between aggregate structure and disease progression (Lu et al., 2013). The intrinsic fluorescent properties of the LCOs also offer the opportunity to use different modes of detection, such as excitation- and emission spectra, as well as fluorescence decays (Magnusson et al., 2014). In addition, these fluorescent dyes also display rather high photo-bleaching thresholds and stability, two parameters that occasionally limit the effectiveness of conventional fluorescent reporter systems (Medintz et al., 2005).

**Figure 1:**
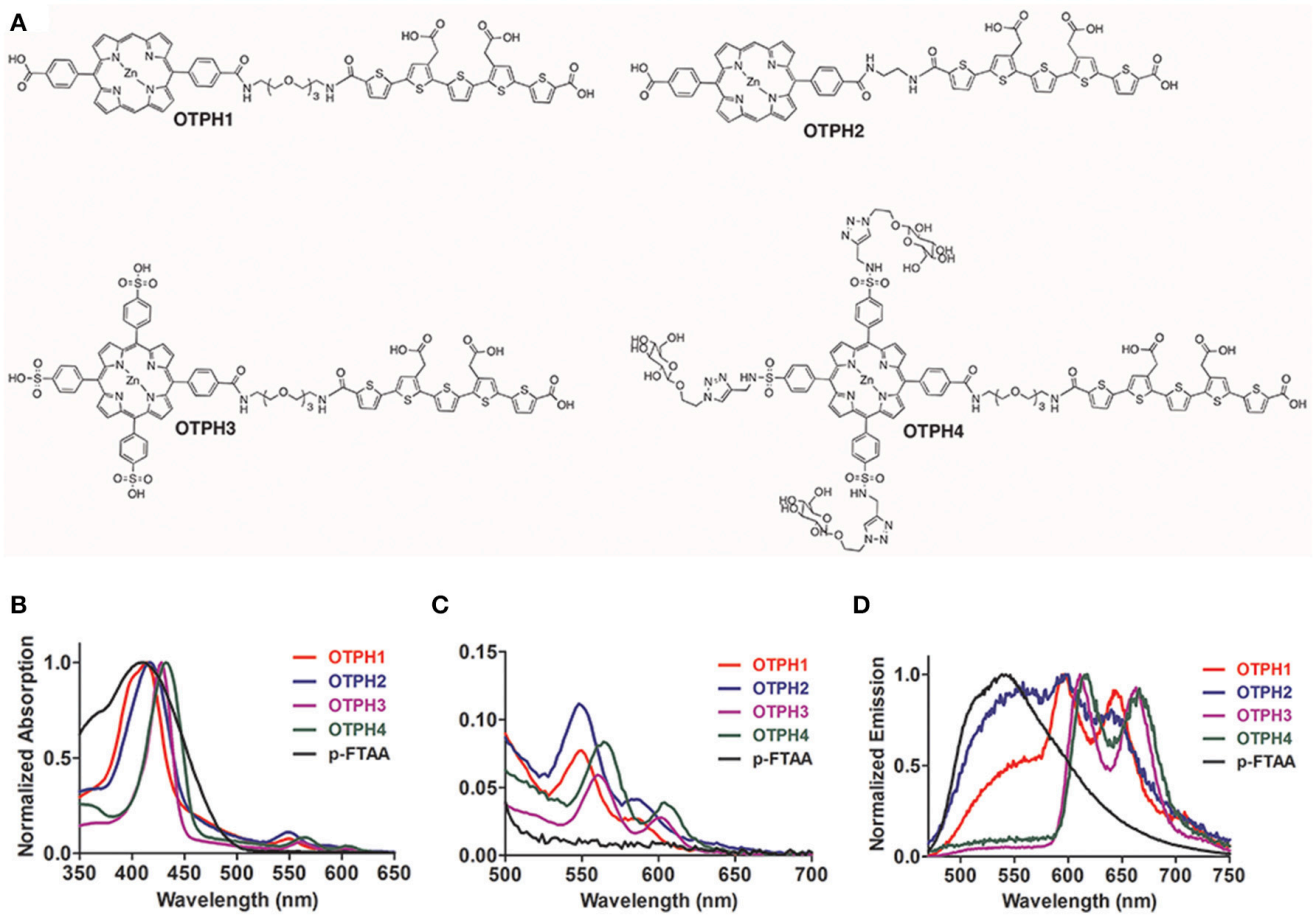
Chemical structures and optical characterization of the oligothiophene porphyrin hybrids (OTPHs). **(A)** Chemical structures of OTPH1, OTPH2, OTPH3, and OTPH4. **(B,C)** Absorption spectra of 3 μM OTPH or LCO dissolved in PBS buffer pH 7.4. **(D)** Emission spectra of 3 μM OTPH or LCO dissolved in PBS buffer pH 7.4.

To enhance the spectral range and the distinct lifetime diversity of the LCOs, we recently reported an anionic pentameric LCO that was conjugated to a porphyrin moiety via a tetraethylene glycol linker (Arja et al., 2013). This hybrid molecule, p-FTAA-porph, contained two optically active moieties and the changes in the interaction between the LCO and the porphyrin upon binding to the Aβ plaque, monitored by emission- and excitation spectra, as well as fluorescence lifetime imaging (FLIM), provided more information about the heterogeneous morphology of the Aβ aggregates than using the LCO alone (Arja et al., 2013). Herein, we present the synthesis of a variety of oligothiophene porphyrin hybrids, OTPHs (Figure 1A) having p-FTAA as the oligothiophene scaffold and varying porphyrin motifs, as well as different spacing between the two optical active moieties. All the OTPHs showed an excellent specificity toward Aβ deposits in a transgenic mouse model with AD-like pathology. In addition, the chemical identity of the porphyrin moiety, as well as the spacing between the two optical active moieties, influenced the OTPHs performance for fluorescent assignment of Aβ deposits. Overall, our studies verified that OTPHs could be utilized for a more precise optical assessment of heterogenous amyloid morphologies and an OTPH with a glycoconjugated porphyrin moiety was identified as an excellent dye for optical assignment of age-dependent Aβ morphotypes. Hence, molecular insight regarding the chemical composition of the OTPHs that will aid in the chemical design of novel OTPHs was obtained.

## Materials and methods

### General procedures

Chemical reagents and solvents were purchased from Sigma-Aldrich and used as received. Microwave heated reactions were run in an Initiator instrument from Biotage. Analytical thin-layer chromatography was performed on Merck silica gel 60 F254 glass- or aluminum backed plates using UV-light (λ = 254 nm) and/or charring with ethanol/sulfuric acid/p-anisaldehyde/acetic acid 90:3:2:1 for visualization. Flash chromatography was performed with silica gel 60 (particles size (0.040–0.063 mm). Analytical liquid chromatography was performed on a Waters system equipped with a Waters 1525 gradient pump, 2998 Photodiode Array Detector, 2424 Evaporative Light Scattering Detector, SQD 2 Mass Detector and an Xbridge® C18 column (4.6 × 50 mm, 3.5 μm). Gradient system: A binary linear gradient of A/B 100:0 → 0:100 over 4 min followed by an additional 2 min at 0:100 was used. (A): 95:5 water:acetonitrile, 10 mM NH_4_OAc; (B) 90:10 acetonitrile:water, 10 mM NH_4_OAc pH 5.0. Preparative liquid chromatography was performed on a Waters system equipped with a 2535 quaternary gradient pump, 2998 Photodiode Array Detector, 2424 Evaporative Light Scattering Detector, SQD 2 Mass Detector and an xselect® phenyl-hexyl column (19 × 250 mm, 5.0 μm). Flow rate 25 mL/min. A binary linear gradient of A (95:5 water:acetonitrile, 10 mM NH_4_OAc pH 5.0) and B (90:10 acetonitrile:water, 10 mM NH_4_OAc pH 5.0) was used. NMR spectra were recorded on a Varian instrument (^1^H 300 MHz, ^13^C 75.4 MHz) with solvent indicated. Chemical shift was reported in ppm on the δ scale and referenced to the solvent peak. IR spectra were recorded on Avatar 330 FT-IR instrument from Thermo Nicolet on Smart Endurance mode. Reported mass data is calculated for average molecular masses according to the mass assignment of macromolecules due to isotopic effects on the centroid mass signal (Barwick et al., 2006).

### Synthesis of OTPHs

***Synthesis of compound 1:*** Methyl 4-formyl benzoate 4.92 g, 30.0 mmol) was dissolved in freshly distilled pyrrole (208 mL, 3.0 mol) and the solution was degassed with a stream of nitrogen for 10 min. The reaction was started by adding InCl_3_ (0.664 g, 3.00 mmol) and continued at room temperature with stirring under nitrogen atmosphere for 1.5 h. Nine grams of NaOH pellets, ground to a coarse powder, was added to the reaction mixture that was continued to be stirred at room temperature for 30 min. The reaction mixture was filtered and the filtrate concentrated *in vacuo*. The remaining crude was thrice co-evaporated with heptane and recrystallized from methanol:water, 10:1. The product was gained as yellowish crystals in two crops in total of 88% yield (7.426 g, 26.49 mmol).

^1^H NMR (300 MHz, CDCl_3_) δ 8.02 − 7.95 (m, 2H), 7.32 − 7.26 (m, 2H), 6.71 (td, *J* = 2.7, 1.5 Hz, 2H), 6.16 (q, *J* = 3.0 Hz, 2H), 5.95 − 5.84 (m, 2H), 5.53 (s, 1H), 3.90 (s, 3H).

^13^C NMR (75 MHz, CDCl_3_) δ 147.27, 129.91, 128.40, 117.51, 109.99, 108.60, 107.50, 52.06, 43.99.

***Synthesis of compound 2:* 1** (0.465 g, 1.66 mmol), dissolved in THF (60 mL), was treated with 1 M aqueous sodium hydroxide solution (7.20 mL, 7.13 mmol) at room temperature for 2 h. The reaction mixture was neutralized with Dowex. After filtration, the solvent was co-evaporated with toluene, which afforded the product as a greenish-beige solid quantitatively.

^1^H NMR (300 MHz, CDCl_3_) δ 8.0 (d, 2H, *J* = 8.22 Hz), 7.92 (bs, 2H), 7.30 (d, 2H, *J* = 8.22 Hz), 6.72 (dd, 2H, *J* = 4.70, 2.93 Hz), 6.17 (dd, 2H, *J* = 5.87, 2.93 Hz), 5.72 (bs, 2H), 5.47 (s, 1H).

^13^C NMR (75 MHz, CDCl_3_) δ 147.0, 131.7, 130.1, 128.6, 117.7, 110.2, 108.8, 107.7, 52.2, 44.2.

***Synthesis of compound 3:*** Phosphoryl chloride (0.30 mL, 3.00 mmol) was added dropwise to DMF (0.11 mL, 1.48 mmol) under argon at 0°C. Immediately a white solid was formed. The reaction was allowed to stand for half an hour and dipyrromethane **1** (0.100 g, 0.357 mmol), dissolved in 1,2-dichloroethane (5.0 mL), was slowly added. The white solid was dissolved and the solution turned purple. The reaction mixture was heated to 35°C and stirred for 25 min. The reaction was quenched by adding ice, followed by aqueous potassium carbonate solution (10% w/v) until the solution was found basic by pH-paper. The organic phase was diluted with DCM, washed twice with water and once with brine, dried over anhydrous magnesium sulfate, and reduced *in vacuo*. FC on silica, eluting with toluene:ethyl acetate, 1:1, afforded the product as an orange-brown foam in 66% yield (0.080 g, 0.236 mmol).

^1^H NMR (300 MHz, CDCl_3_) δ 10.44 (s, 2H), 9.22 (s, 2H), 8.08 − 7.95 (m, 2H), 7.42 − 7.32 (m, 2H), 6.87 (dd, *J* = 3.9, 2.3 Hz, 2H), 6.04 (ddd, *J* = 3.8, 2.5, 0.7 Hz, 2H), 5.63 (s, 1H), 3.91 (s, 3H).

^13^C NMR (75 MHz, CDCl_3_) δ 179.0, 144.1, 140.4, 132.8, 130.3, 128.5, 122.0, 111.7, 52.2, 44.4.

***Synthesis of compound 4:*** Formylated dipyrromethane **3** (0.090 g, 0.268 mmol) was dissolved in THF (1 mL) whereupon *n*- propane amine (440 μL, 5.35 mmol) was added. The reaction mixture was stirred at room temperature for 3 h and concentrated to dryness.

^1^H NMR (300 MHz, CDCl_3_) δ 7.97 (d, *J* = 8.5 Hz, 2H), 7.93 (t, *J* = 1.2 Hz, 2H), 7.27 (d, *J* = 8.1 Hz, 2H), 6.34 (d, *J* = 3.6 Hz, 2H), 5.88 (dd, *J* = 3.6, 0.7 Hz, 2H), 5.45 (s, 1H), 3.90 (s, 3H), 3.40 (td, *J* = 7.0, 1.2 Hz, 4H), 1.60 (h, *J* = 7.3 Hz, 4H), 0.89 (t, *J* = 7.4 Hz, 6H).

***Synthesis of compound 5:* 4** (0.112 g, 0.268 mmol assuming quantitative yield) and **2** (0.071 g, 0.268 mmol) were dissolved in ethanol (30 mL) whereupon Zn(OAc)_2_ (0.550 g, 3.00 mmol) was added. The solution, that turned immediately deep purple, was refluxed for 20 h. The solvent was reduced under vacuum and the crude product purified on silica with mobile phase toluene:ethyl acetate, 9:1, with 0.1% acetic acid. As a result of ethanol reflux some of the methyl ester was transesterified to the corresponding ethyl ester. The mixed methyl/ethyl ester porphyrin was obtained as purple-red solid in 30% yield (0.051 g, 0.081 mmol).

^1^H NMR (300 MHz, CDCl_3_/CD_3_OD) δ 10.25 (s, 2H), 9.39 (d, *J* = 4.7 Hz, 4H), 9.00 (two d, *J* = 4.11 Hz and 4.7 Hz, 4H), 8.46 (d, *J* = 6.5 Hz, 4H), 8.35 (app. m, 4H), 4.60 (q, *J* = 7.0 Hz, 2H), 4.10 (s, 3H), 1.55 (t, *J* = 7.0 Hz, 2H).

The mixed methyl-ethyl ester porphyrin, with a carboxylic acid on the other side, (0.040 g, 0.064 mmol) was dissolved in DMF (1.0 mL) whereupon EDC (0.018 g, 0.096 mmol), NHS (0.011 g, 0.096 mmol) and DIPEA (33 μL, 0.191 mmol) were added to it. After stirring the reaction mixture for 7.5 h at room temperature the same amounts of EDC, NHS and DIPEA were added to the solution as the TLC analysis showed incomplete reaction. After the total of 23 h the reaction solution was diluted with chloroform and washed twice with brine. The organic phase was dried over anhydrous magnesium sulfate and reduced *in vacuo*. The residue was chromatographed on silica with the mobile phase toluene:ethyl acetate, 6:1. Compound **5** was obtained in 52% yield (0.024 g, 0.033 mmol).

^1^H NMR (300 MHz, CDCl_3_) δ 10.35 (s, 2H), 9.47 (d, *J* = 4.6 Hz, 2H), 9.45 (d, *J* = 4.6 Hz, 2H), 9.09 (d, *J* = 4.6 Hz, 2H), 9.06 (d, *J* = 4.6 Hz, 2H), 8.58 (d, *J* = 8.5 Hz, 2H), 8.47 (d, *J* = 8.5 Hz, 2H), 8.42 (d, *J* = 8.4 Hz, 2H), 8.34 (d, *J* = 8.6 Hz, 2H), 4.59 (q, *J* = 7.1 Hz, 1H), 4.13 (s, 1H), 3.02 (s, 4H), 1.57 (t, *J* = 7.1 Hz, 2H). ^13^C NMR (75 MHz, DMSO-*d*_6_) δ 170.5, 170.1, 149.9, 149.1, 149.1, 148.7, 148.7, 148.5, 147.4, 135.4, 134.8, 132.6, 132.5, 131.6, 131.5, 130.7, 130.1, 128.9, 128.4, 127.5, 123.4, 118.0, 117.1, 106.5, 61.0, 25.7, 25.6, 14.4.

***Synthesis of compound 6:*** Methyl 4-formylbenzoate (0.821 g, 5.00 mmol) was dissolved in DCM (200 ml), where benzaldehyde (1.59 mL, 15.00 mmol), pyrrole (1.39 mL, 20 mmol), and ground sodium chloride (0.117 g, 2.00 mmol) were added to. The solution was degassed with a stream of nitrogen under constant stirring at room temperature for 10 min. Boron trifluoride etherate (247 μL, 2.00 mmol) was added and the reaction mixture was stirred for another 30 min until methyl 4-formylbenzoate no longer was evident by TLC analysis. DDQ (3.40 g, 15.00 mmol) was added and the reaction was let continue for an hour. Triethyl amine (2.00 mL) was added to the reaction mixture and the solvent was reduced *in vacuo*. The crude mixture was filtered over a short column of silica eluting with DCM followed by purification on FC on silica eluting with DCM:heptane, 1:1. Methyl mono-(p-carboxy)-tetraphenylporphyrin (Me-pcTPP) **6** was isolated from the second purple band on silica column and gave deep purple crystals in 19% yield (0.629 g, 0.935 mmol).

^1^H NMR (300 MHz, CDCl_3_) δ 8.88 − 8.82 (m, 6H), 8.78 (d, *J* = 4.8 Hz, 2H), 8.44 (d, *J* = 8.3 Hz, 2H), 8.30 (d, *J* = 8.4 Hz, 2H), 8.24 − 8.18 (m, 6H), 7.78 − 7.72 (m, 8H), 4.11 (s, 3H),−2.77 (s, 2H).

^13^C NMR (75 MHz, CDCl_3_) δ 167.3, 147.1, 144.1, 142.1, 142.1, 134.6, 134.5, 129.6, 127.9, 127.8, 126.7, 120.4, 118.5, 52.4.

***Synthesis of compound 7:* 6** (0.337 g, 0.500 mmol) was dissolved in chlorosulfonic acid (6 mL) and the solution was stirred at room temperature for 1.5 h. The solution was diluted with dichloromethane (200 mL) and washed carefully with ice-cold water (3x) and brine. The organic phase was dried over anhydrous sodium sulfate and evaporated to dryness. The chlorosulfonated intermediate was suspended in water (50 mL), sonicated for 3 min and subjected to hydrolysis at 80°C for 12 h. LC-MS analysis indicated a complete conversion of the sulfonyl chlorides to sulfonates together with some hydrolysis of the methyl ester. The solvent was evaporated *in vacuo*. The green solid was dissolved in water (50 mL) and aqueous sodium hydroxide (1 M, 5 mL) was added. The reaction mixture was refluxed for 1 h, cooled to room temperature and neutralized with Dowex. The solution was filtered and concentrated *in vacuo*. The product was obtained without further purification in quantitative yield (0.447 g, 0.498 mmol).

^1^H NMR (300 MHz, D_2_O) δ 8.11 − 7.91 (m, 8H), 7.87 (d, *J* = 7.2 Hz, 6H), 7.11 (d, *J* = 8.0 Hz, 1H), 6.87 (d, *J* = 7.9 Hz, 8H). ^13^C NMR (75 MHz, D_2_O) δ 174.9, 143.1, 142.8, 142.7, 142.0, 141.8, 141.8, 135.3, 134.9, 134.6, 127.1, 123.8, 123.6, 117.3, 117.2.

***Synthesis of compound 8:* 6** (0.200 g, 0.297 mmol) was treated with chlorosulfonic acid (5.0 mL) for 30 min. The reaction mixture was diluted with DCM and washed with ice-water (3x) and brine. The organic phase was dried over anhydrous sodium sulfate, filtered and concentrated *in vacuo*. The obtained chlorosulfonated intermediate was directly dissolved in DCM (20 mL) whereupon propargylamine (114 μL, 1.78 mmol) and DIPEA (373 μL, 2.68 mmol) were added. The reaction mixture was stirred at room temperature for an hour. The solution was diluted with DCM and washed with water and brine. The organic phase was dried over anhydrous sodium sulfate, filtered and concentrated *in vacuo*. The crude was purified on silica FC eluting with DCM:MeOH, 30:1, followed by 20:1. The isolated product was subjected to metallation by zinc(II) by dissolving the porphyrin in DCM (20 mL) followed by addition of zinc acetate dihydrate (91.4 mg, 0.416 mmol) dissolved in a minimal volume of methanol. The reaction mixture was stirred at room temperature for approximately 2 h until the metallation process was completed and then diluted with DCM. The solution was washed with water and brine, dried over sodium sulfate and concentrated *in vacuo*. Zinc-porphyrin complex **8** was obtained as deep purple solid in 40% yield (0.129 g, 0.119 mmol).

H NMR (300 MHz, Acetone-*d*_6_, CD_3_OD) δ 8.76 (bs, 8H), 8.37 (d, *J* = 8.2 Hz, 2H), 8.29 − 8.13 (m, 14H), 4.11 (s, 3H), 4.05 − 4.01 (m, 6H), 2.43 (app. dt, *J* = 4.9, 2.5 Hz, 3H). ^13^C NMR (75 MHz, CDCl_3_) δ 171.4, 150.3, 150.0, 143.9, 143.9, 138.5, 138.3, 133.6, 131.8, 129.5, 129.4, 123.5, 122.6, 122.5, 110.0, 82.4, 76.4, 56.1, 36.2.

***Synthesis of compound 9:* 8** (0.184 g, 0.169 mmol) and lithium chloride (0.180 g, 4.25 mmol, 25 eq) were dissolved in DMF (3 mL) in a microwave oven vial. The solution was subjected to microwave irradiation for a total of 2 h at 160°C. The demethylation was followed by LC-MS analysis. Upon completion, the reaction mixture was diluted in ethyl acetate and washed with aqueous HCl (1 M), water and brine. The organic phase was dried over anhydrous sodium sulfate and evaporated *in vacuo*. The crude product was purified by FC on silica eluting with DCM:MeOH, 30:1, and 0.5% formic acid. The free acid porphyrin was isolated in 77% yield (0.141 g, 0.131 mmol) as deep purple solid. The porphyrin from the previous step (0.065 g, 0.061 mmol) and acetylated azidoethyl β-glucoside (0.091 g, 0.218 mmol) were dissolved in THF (3 mL) in a microwave oven vial whereupon tert-butanol (1 mL), water (0.5 mL), copper(II) sulfate (4.83 mg, 0.030 mmol) and sodium L(+)-ascorbate (12.00 mg, 0.061 mmol) were added. The solution was subjected to microwave irradiation for 3 min at 85°C. The reaction mixture was diluted in ethyl acetate and washed with water and brine. The organic phase was dried over anhydrous sodium sulfate and evaporated *in vacuo*. The crude was purified on silica column eluting with DCM:MeOH, 20:1, and 0.5% formic acid. Triglycosylated **9** was isolated in 59% yield (0.083 g, 0.036 mmol) as purple solid.

^1^H NMR (300 MHz, DMSO-*d*_6_) δ 8.91 − 8.79 (m, 8H), 8.52 (t, *J* = 5.8 Hz, 3H), 8.44 − 8.30 (m, 10H), 8.21 (d, *J* = 7.9 Hz, 6H), 8.02 (s, 3H), 5.10 (2 dd, *J* = 9.3, 2.5 Hz, 3H), 4.85 (app. t, *J* = 9.7 Hz, 3H), 4.79 − 4.68 (m, 6H), 4.64 − 4.50 (m, 6H), 4.40 (d, *J* = 4.8 Hz, 6H), 4.12 − 3.83 (m, 12H), 3.78 − 3.64 (m, 3H), 1.96 (s, 9H), 1.95 (s, 9H), 1.91 (s, 18H). ^13^C NMR (75 MHz, DMSO-*d*_6_) δ 170.0, 169.5, 169.2, 169.0, 149.2, 149.1, 149.0, 149.0, 146.5, 146.5, 143.4, 143.3, 139.7, 134.6, 131.9, 124.9, 124.9, 124.9, 123.8, 119.1, 119.1, 99.2, 71.9, 70.6, 70.6, 67.9, 67.5, 61.4, 49.3, 38.4, 20.4, 20.3, 20.2, 20.2.

***Synthesis of compound 11:*** LCO **10** (Åslund et al., 2009) (190.0 mg, 0.271 mmol) was dissolved in DMF (4 mL) together with the asymmetric TEG linker (Supplementary Material Scheme S1) (160.0 mg, 0.407 mmol) whereupon HATU (154.6 mg, 0.407 mmol) and triethyl amine (113 μL, 0.813 mmol) were added to it. The reaction mixture was stirred at room temperature for 3.5 h. The solution was diluted with ethyl acetate and washed with water and brine. The organic phase was dried over anhydrous sodium sulfate and evaporated *in vacuo*. The crude was purified with semi-preparative HPLC on a reversed phase column. Compound **11** was isolated in 0.173 g (0.161 mmol, 59%), the corresponding mono-Boc-protected compound was isolated in 0.071 g (0.073 mmol, 25%).

^1^H NMR (300 MHz, CDCl_3_) δ 7.62 (d, *J* = 3.9 Hz, 1H), 7.47 (d, *J* = 3.9 Hz, 1H), 7.23 (s, 1H), 7.20 (s, 3H), 7.12 (d, *J* = 3.9 Hz, 2H), 6.92 (t, *J* = 4.9 Hz, 1H), 3.78 (d, *J* = 7.0 Hz, 13H), 3.68 − 3.61 (m, 13H), 1.59 (s, 9H), 1.50 (s, 18H). ^13^C NMR (75 MHz, CDCl_3_) δ 170.9, 161.6, 161.2, 152.7, 142.4, 140.9, 137.9, 135.5, 135.4, 135.3, 134.1, 133.5, 133.0, 132.7, 131.3, 131.3, 128.6, 128.1, 127.9, 127.7, 127.6, 124.1, 124.0, 82.4, 82.0, 70.6, 70.3, 70.2, 69.8, 69.3, 67.1, 52.4, 45.2, 39.8, 34.8, 28.2, 28.1.

***Synthesis of compound 12:*** LCO **10** (Åslund et al., 2009) (10.0 mg, 14.3 μmol) was dissolved in DMF (5 mL) together with N-Boc-ethylenediamine (6.86 mg, 42.8 μmol) where after HATU (16.28 mg, 42.80 μmol) and triethylamine (12.0 μL, 85.61 μmol) were added to it. The reaction mixture was stirred at room temperature for 2 h and the solvent was evaporated under a flow of nitrogen. After purification on preparative HPLC and lyophilization compound **12** was isolated as orange solid in 58% yield (7.0 mg, 8.28 μmol).

^1^H NMR (300 MHz, DMSO-*d*_6_) δ 8.56 (t, *J* = 5.6 Hz, 1H), 7.69 (d, *J* = 4.0 Hz, 1H), 7.66 (s, 1H), 7.49 (s, 1H), 7.42 − 7.38 (m, 2H), 7.37 (d, *J* = 3.9 Hz, 1H), 7.35 − 7.29 (m, 2H), 6.90 (t, *J* = 5.8 Hz, 1H), 3.88 (s, 4H), 3.67 (s, 6H), 3.31 (m, 2H), 3.09 (q, *J* = 6.4 Hz, 2H), 1.54 (s, 9H), 1.38 (s, 9H). ^13^C NMR (75 MHz, DMSO-*d*_6_) δ 170.9, 170.9, 161.2, 160.7, 156.1, 142.1, 139.7, 139.5, 135.1, 134.8, 134.7, 134.6, 134.0, 133.5, 132.8, 132.8, 132.6, 131.9, 130.3, 129.5, 128.3, 128.2, 125.4, 125.4, 110.0, 82.3, 78.1, 52.5, 34.6, 28.7, 28.3.

***Synthesis of OTPH1:* 11** (0.118 g, 0.114 mmol) was dissolved in DCM (8 mL) and treated with TFA (2 mL) for 40 min. Methanol (10 mL) was added and the solution was stirred for another 5 min. The solvent was evaporated *in vacuo* and residual TFA co-evaporated with toluene (3x) to give **13** (0.095 g, 0.114 mmol) in quantitative yield as a red solid without further purification. The deprotected product was dissolved in DMF (7 mL) and added to a solution of NHS-activated porphyrin **5** (0.083 g, 0.114 mmol) in DMF (7 mL). DIPEA (20 μL, 0.114 mmol) was added and the reaction mixture was stirred for 48 h. The solvent was co-evaporated with toluene (3x). FC on silica gave the protected LCO-porphyrin conjugate in 60% yield (0.099 g, 0.069 mmol) that was treated with sodium hydroxide (1 M, aq., 0.413 mL, 0.413 mmol) in dioxane (12 mL) under reflux for 3 h. The solution was neutralized with Dowex and the solvent reduced *in vacuo*. The crude was purified by reverse-phase HPLC to yield **OTPH1** as deep red crystals in 73% yield (0.070 g, 0.050 mmol). Tests for water solubility were done on both the protonated acids and sodium carboxylates. The former was insoluble in water while the sodium salt was water-soluble.

*m*/*z*: [M + H]^+^ calcd for C_68_H_52_N_6_O_13_S_5_Zn, 1385.2; found, 1385.1. IR 1592, 1520, 1455, 1390, 1319, 1058, 1026, 994, 880, 773 cm^−1^.

^1^H NMR (300 MHz, DMSO-*d*_6_, δ): 10.37 (s, 2H, Ar H), 9.50 (d, *J* = 4.5 Hz, 4H, Ar H), 8.94 (app t, *J* = 4.2 Hz, 4H, Ar H), 8.89 (t, *J* = 5.4 Hz, 1H, CONH), 8.66 (t, *J* = 5.3 Hz, 1H, CONH), 8.39 (d, *J* = 8.2 Hz, 2H, Ar H), 8.36 − 8.25 (m, 6H, Ar H), 7.73 (d, *J* = 3.9 Hz, 1H, Ar H), 7.31 (s, 1H, Ar H), 7.30 − 7.26 (m, 3H, Ar H), 7.24 (d, *J* = 3.8 Hz, 1H, Ar H), 7.22 (s, 1H, Ar H), 7.15 (d, *J* = 3.7 Hz, 1H, Ar H), 3.74 − 3.43 (m, 20H, CH_2_). ^13^C NMR (75 MHz, DMSO-*d*_6_, δ): 172.5, 169.1, 167.0, 164.8, 161.3, 149.4, 149.4, 149.4, 146.3, 145.9, 144.8, 140.3, 139.0, 138.9, 138.1, 135.5, 135.1, 134.9, 134.8, 133.7, 133.7, 133.1, 132.7, 132.0, 131.9, 131.3, 130.2, 130.0, 129.6, 129.3, 128.8, 128.6, 128.0, 127.7, 127.4, 126.0, 125.0, 124.5, 118.8, 118.5, 106.7, 70.3, 70.2, 70.1, 69.4, 69.4, 57.9, 41.4.

***Synthesis of OTPH2:* 12** (25.1 mg, 29.8 μmol) was dissolved in DCM (4 mL) and treated with TFA (1 mL) for 30 min. Methanol (2 mL) was added and the solution was stirred for another 5 min. The solvent was evaporated *in vacuo* and residual TFA co-evaporated with toluene to give **14** as a red solid. The resulting amine was used in the following amide coupling reaction without further purification. **14** was dissolved in DMF (2 mL) and added to a solution of NHS-activated porphyrin **5** (18.0 mg, 24.8 μmol) in DMF (2 mL). DIPEA (13 μL, 75.0 μmol) was added and the reaction mixture was stirred for 12 h. The solution was diluted with ethyl acetate:THF mixture, 1:1, and washed with water and brine. The organic phase was dried over sodium sulfate and evaporated to dryness. Purification on FC, eluting with DCM:MeOH, 30:1, with 0.05% formic acid, gave the methyl ester protected intermediate. The demethylation of the remaining esters was done with sodium hydroxide (1 M, aq., 149.0 μL, 149.0 μmol) in THF (3 mL) at room temperature for 3 h. The solution was neutralized with Dowex and the solvent reduced *in vacuo*. The deep red product showed poor solubility in several solvent and was purified by washing with water and methanol. The solid was dried yielding **OTPH2** in 29% (9.0 mg, 7.2 μmol).

*m*/*z*: [M + H]^+^ calcd for C_62_H_41_N_6_O_10_S_5_Zn, 1255.7; found, 1255.6. IR 3427, 1436, 1407, 1018, 952, 699, 668 cm^−1^. ^1^H NMR (300 MHz, DMSO-*d*_6_) δ 10.34 (s, 2H), 9.46 (d, *J* = 4.5 Hz, 4H), 8.94 (s, 1H), 8.88 (dd, *J* = 4.4, 2.3 Hz, 4H), 8.80 (s, 1H), 8.44 − 8.21 (m, 8H), 7.76 (d, *J* = 3.9 Hz, 1H), 7.69 (d, *J* = 4.1 Hz, 1H), 7.62 (q, *J* = 5.1 Hz, 2H), 7.42 (s, 1H), 7.36 (q, *J* = 3.2 Hz, 2H), 7.23 (s, 1H), 3.59 (d, *J* = 1.6 Hz, 5H). Poor solubility of the compound hindered ^13^C NMR to be recorded.

***Synthesis of OTPH3:* 11** (24.0 mg, 22.2 μmol) was dissolved in DCM (4 mL) and treated with TFA (1 mL) for 20 min. Methanol (2) mL) was added and the solution was stirred for another 5 min. The solvent was evaporated *in vacuo* and residual TFA co-evaporated with toluene (3x) to give **13** as a red solid. **13** was used in the following amide coupling reaction without any further purification. Porphyrin 7 (24.0 mg, 26.6 μmol) together with 13 was dissolved in DMF (5 mL). HATU (25.3 mg, 66.6 μmol) and DIPEA (12.0 μL, 66.6 μmol) were added to this solution and the reaction mixture was let to stir at room temperature for an hour. The solvent was concentrated *in vacuo* and the crude was purified on HPLC. The isolated protected product was dissolved in THF-H2O-mixture (10 mL) whereupon aqueous sodium hydroxide solution (1 M, 1.0 mL) was added to it. The ester deprotection proceeded for 1.5 h at room temperature. The solution was neutralized with Dowex and filtered. Metallation with zinc was conducted by adding Zn(OAc)2(H2O)2 (39.6 mg, 180.5 μmol) to the filtrate. The reaction was monitored with analytical HPLC-MS and upon completion the reaction solution was concentrated to dryness. Purification on preparative HPLC, followed by lyophilization, yielded compound OTPH3 as purple solid in 78% (30.0 mg, 17.3 μmol).

*m*/*z*: [M + H]^+^ calcd for C_79_H_61_N_6_O_20_S_8_Zn, 1736.2; found, 1736.7. IR 3417, 1594, 1455, 1376, 1203, 1126, 1040, 1007, 995, 880, 795, 740 cm ^−1^. ^1^H NMR (300 MHz, DMSO-*d*_6_) δ 8.84 (t, *J* = 5.5 Hz, 1H), 8.81 − 8.72 (m, 8H), 8.62 (t, *J* = 5.8 Hz, 1H), 8.24 (d, *J* = 1.7 Hz, 4H), 8.12 (dd, *J* = 8.3, 1.5 Hz, 6H), 8.05 − 7.94 (m, (m, 6H), 7.71 (d, *J* = 4.0 Hz, 1H), 7.43 (d, *J* = 3.9 Hz, 1H), 7.38 − 7.27 (m, 3H), 7.27 (s, 1H), 7.24 − 7.13 (m, 2H), 3.75 − 3.46 (m, 20H). ^13^C NMR (75 MHz, DMSO-*d*_6_) δ 167.0, 149.7, 149.7, 149.4, 147.8, 146.0, 143.2, 134.5, 134.0, 132.2, 131.9, 125.9, 124.2, 120.6, 120.5, 119.8, 70.3, 70.2, 70.2, 69.5, 69.4, 21.6.

***Synthesis of OTPH4:*** Compound **9** (56.0 mg, 24.1 μmol) was dissolved in DMF (5 mL). NHS (8.3 mg, 72.2 μmol) was added, followed by EDC (6.9 mg, 36.1 μmol). The reaction mixture was stirred at room temperature for 18 h at which point full conversion of the acid into the succinate ester was confirmed by LC-MS analysis. The reaction mixture was diluted in ethyl acetate and washed with water and brine. The organic phase was dried over sodium sulfate and evaporated to dryness. Meanwhile, amine **13** was produced from 11 (45.0 mg, 46.1 μmol) by treating it in DCM:TFA, 4:1, (5 mL) for 30 min, followed by evaporation of solvents. 13 was dissolved in DMF (2 mL) and added to the solution of NHS-activated 9 in DMF (3 mL) followed by TEA (10.0 μL, 71.2 μmol). The reaction mixture was stirred at room temperature for 24 h, diluted with ethyl acetate and washed with 0.5 M HCl (aq), water and brine. The organic phase was dried over anhydrous sodium sulfate and concentrated *in vacuo*. The crude was purified by FC on silica eluting with DCM:MeOH, 30:1, 0.5% formic acid, followed by DCM:MeOH, 20:1, 0.5% formic acid. The isolated product was subjected to deprotection of esters by dissolving it in a mixture of THF (5 mL) and water (5 mL) followed by addition of aqueous sodium hydroxide (1 M, 0.6 mL, 0.6 mmol). After an hour the reaction solution was neutralized with Dowex, filtered and concentrated *in vacuo*. The crude was purified by preparative HPLC yielding OTPH4 in 19% yield (12.0 mg, 4.63 μmol) as reddish solid.

m/z: [M + H]+ calcd for C112H115N18O35S8Zn, 2595.1; found, 2595.4. IR 3306, 2901, 2367, 1591, 1457, 1370, 1329, 1159, 1075, 1049, 1026, 994, 796, 965, 733 cm^−1^. ^1^H NMR (300 MHz, DMSO-*d*_6_) δ 8.84 − 8.78 (m, 1H), 8.76 − 8.65 (m, 8H), 8.49 (t, *J* = 5.5 Hz, 1H), 8.34 − 8.17 (m, 11H), 8.17 − 8.06 11H), 7.59 (d, *J* = 3.9 Hz, 1H), 7.10 (d, *J* = 3.7 Hz, 1H), 7.04 (d, *J* = 3.8 Hz, 3.8 Hz, 1H), 7.02 − 6.98 (m, 2H), 6.89 (d, *J* = 3.8 Hz, 1H), 6.85 (d, *J* = 3.6 Hz, 1H), 6.79 (s, 1H), 4.50 (d, *J* = 5.0 Hz, 6H), 4.31 (d, *J* = 2.8 Hz, 6H), 4.13 (d, *J* = 7.8 Hz, 4H), 4.06 − 3.90 (m, 3H), 3.80 (dd, *J* = 10.5, 5.1 Hz, 3H), 3.66 − 3.43 (m, 19H), 3.32 (dd, *J* = 11.7, 5.1 Hz, 5H), 3.15 − 2.85 (m, 15H). ^13^C NMR (75 MHz, DMSO-*d*_6_) δ 149.0, 149.0, 146.5, 143.3, 141.6, 139.7, 136.2, 134.5, 131.8, 124.9, 124.5, 119.0, 109.5, 102.9, 77.0, 76.6, 73.3, 70.0, 69.8, 69.7, 67.3, 61.0, 49.7, 21.3.

### Optical characterization of the OTPH

Stock solutions of dyes (1.5 mM corresponding sodium salt in de-ionized water for OTPH1, OTPH3 and OTPH4, or de-ionized water with 10% DMSO for OTPH2) were diluted to 3 μM in a PBS buffer (10 mM phosphate, 150 mM NaCl pH 7.4 prepared from PBS (Phosphate-Buffered Saline) Tablets (Invitrogen, USA)). Absorption- and emission spectra of the molecules were collected using an Infinite M1000 Pro microplate reader (Tecan, Männedorf, Switzerland).

### Tissue staining with OTPHs

Animal handling was carried out in accordance with relevant guidelines of breeding and keeping transgenic animals as well as culling and collection of tissue for *ex vivo* experiments. Cryosections (20 μm) of tissue from APPPS1 transgenic mice with Aβ pathology were fixed with ethanol for 10 min, re-hydrated with de-ionized water, incubated with PBS for 10 min and then stained for 30 min at room temperature with p-FTAA, OTPH1, OTPH2, OTPH3 or OTPH4. All of the probes were diluted 1:500 in PBS buffer from a 1.5 mM stock solution (de-ionized water for OTPH1, OTPH3 and OTPH4, or de-ionized water with 10% DMSO for OTPH2). After rinsing with PBS buffer three times, the sections were mounted with Dako fluorescence mounting medium (Dako Cytomation, Glostrup, Denmark). The medium was allowed to solidify for 3 h before the rims were sealed with nail polish.

### Spectral- and fluorescence lifetime imaging of stained tissue sections

Spectral images of stained tissue sections were acquired on an inverted Zeiss (Axio Observer.Z1) LSM 780 microscope equipped with a 32 channel QUASAR GaAsP spectral array detector. For all imaging a Plan-Apochromat 40 × /1.3 Oil DIC objective lens was used. Excitation was done by simultaneous excitation of three laser lines; an argon laser at 458 nm and 488 nm, and a DPSS (561-10) laser at 561 nm. Emission spectra were collected between 416 and 687 nm. Fluorescence lifetime images were acquired using the same microscope system as for the spectral imaging of stained tissue sections. In this setup the emitted photons were routed through the Direct coupling (DC) confocal port of the Zeiss LSM 780 scanning unit and detected by a Becker & Hickl HPM-100-40 hybrid photomultiplier tube (PMT). The data were recorded by a Becker & Hickl Simple-Tau 152 system (SPC-150 TCSPC FLIM module) with the instrument recording software SPCM version 9.42 in the FIFO image mode using 256 time channels (Becker & Hickl GmbH, Berlin, Germany). For all acquisitions a Plan-Apochromat 40 × /1.3 Oil DIC objective lens was used, and the pinhole was set to 20.2 μm. For excitation at 490 nm and 565 nm the pulsed tunable In Tune laser with a repetition rate of 40 MHz were used. Data was analyzed in SPCImage version 3.9.4 (Becker & Hickl GmbH, Berlin, Germany).

## Results and discussion

### Synthesis of the OTPHs

**Scheme 1:**
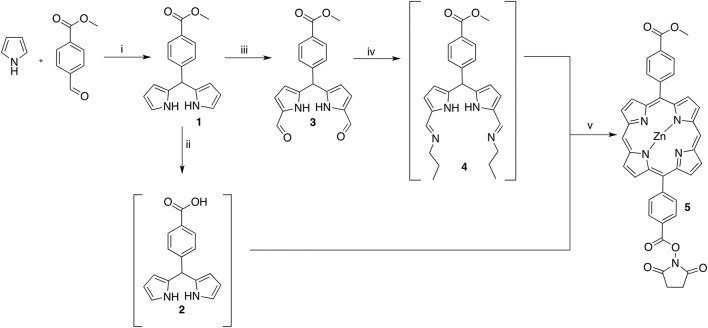
Reagents and conditions: (i) 1. InCl_3_, r.t. 2. NaOH, r.t. 88% (ii) NaOH (aq), THF, r.t. quant. (iii) 1. POCl_3_, DMF, 1,2-dichloroethane, 0–35°C; 2. K_2_CO_3_, H_2_O, 0°C; 66% (iv) propan-1-amine, THF, r.t. quant. (v) 1. Zn(OAc)_2_(H_2_O)_2_, EtOH, reflux; 2. NHS, EDC, DIPEA, DMF, r.t. 16%.

**Scheme 2:**
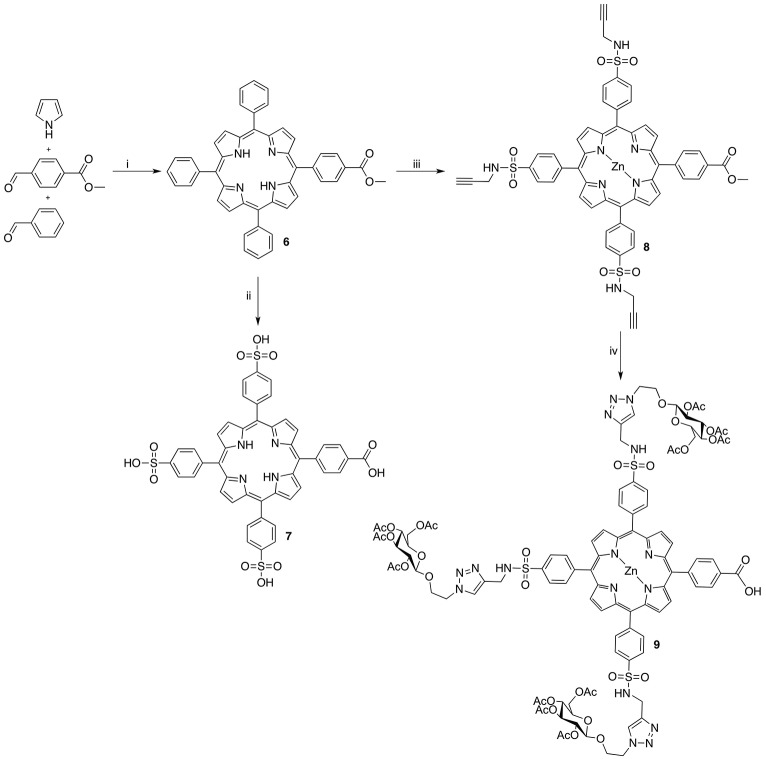
Reagents and conditions: (i) 1. BF_3_•OEt_2_, NaCl, DCM, r.t. 2. DDQ, r.t. 3. TEA, r.t. 19% (ii) 1. HSO_3_Cl, r.t. 2. H_2_O, 80°C; 3. NaOH, r.t., quant. (iii) 1. HSO_3_Cl, r.t. 2. Propargylamine, DIPEA, DMF, r.t. 3. Zn(OAc)_2_(H_2_O)_2_, DCM, MeOH, r.t., 40% over 2 steps; (iv) 1. LiCl, DFM, MW, 160°C, 77%. 2. Acetylated azidoethyl glucoside, CuSO_4_, sodium L(+)-ascorbate, THF, t-BuOH, H_2_O, MW, 85°C, 59%.

In order to generate a small library of OTPHs with distinct porphyrin moieties three different porphyrins were used in building the conjugates – **5**, **7**, and **9** (Schemes 1, 2). Porphyrin **5** was synthesized according to Fan et al. (2005) using dipyrromethane building blocks **2** and **4**. For orthogonally functionalized **5**, a porphyrin featuring a methyl ester and a free acid was produced and directly turned into the corresponding succinate ester for an easier purification. A yield of 16% of compound **5** was considered satisfactory (Scheme 1). For the synthesis of porphyrin building blocks **7** and **9**, methyl mono(p-carboxy)tetraphenylporphyrin) **6** was used as the starting porphyrin (Scheme 2). **6** was easily accessible using the Lindsey's method of a one-pot two-step reaction of pyrrole, benzaldehyde and methyl 4-formylbenzoate under the BF_3_∙OEt_2_ catalysis followed by DDQ oxidation. Also from Lindsey, a modification of the reaction using 0.1 M NaCl solution in DCM was employed, as the reaction medium improved the isolated yield of **6** to 19% (Li et al., 1997).

To generate porphyrin **7**, **6** was treated with chlorosulfonic acid producing porphyrin tris(sulfonyl chloride) that was hydrolyzed to the target compound in quantitative yield. To obtain compound **9**, a high-yielding and exclusively regioselective alkyne-azide cycloaddition (Huisgen et al., 1967) was chosen for the coupling reaction to achieve glycoconjugation of glucose on three sites of the porphyrin. For this reason, tris-alkyne functionalized and zinc-metallated **8** was prepared by letting chlorosulfonated intermediate **7** react with propargylamine in the presence of DIPEA. After the subsequent metalation with zinc, using zinc acetate dihydrate, **8** was obtained in 40% yield. Before executing the click-reaction, methyl ester on **8** was hydrolyzed to the corresponding acid for the later coupling reaction at it. A mild and selective Krapcho demethylation (Krapcho et al., 1967) was applied under microwave irradiation (Wu et al., 2009; Mason and Murphree, 2013) which left the sulfonamide bonds intact. The glycosylation protocol used Sharpless' copper(I) catalyzed modification of the Huisgen 1,3-cycloaddition (Rostovtsev et al., 2002) under microwave activation. The conditions were adapted from Maillard and coworkers (Garcia et al., 2011) offering a mild and highly efficient conjugation at only 5 min of reaction time at 85°C in a sealed vial under microwave irradiation. The isolated yield of compound **9** was 59%.

**Scheme 3:**
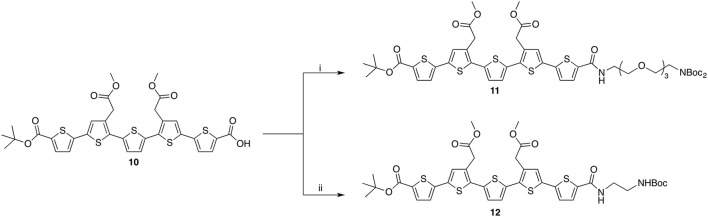
Reagents and conditions: (i) TEG-linker, HATU, TEA, DMF, r.t., 59%; (ii) ethylene mono-Boc diamine, HATU, DIPEA, DMF, r.t. 58%.

As the oligothiophene moiety, the negatively charged LCO, p-FTAA, was selected. For the selective mono-conjugation asymmetrically protected precursor **10** was synthesized according to the published literature (Nordeman et al., 2016). Compound 10 was coupled to two linker molecules of different lengths producing compound 11 and 12 (Scheme 3). For 11, orthogonally functionalized tetraethylene glycol (TEG, Supplementary Material Scheme S1) linker was used whilst conjugation to ethylene mono-Boc diamine rendered compound 12. The two conjugations utilized HATU mediated amide bond formation between the acid on the LCOs and the amine on the linkers.

**Scheme 4:**
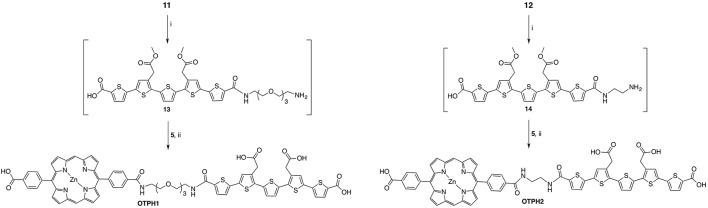
Reagents and conditions: (i) DCM:TFA, 4:1, r.t. (ii) DIPEA, DMF, r.t., 73% for OTPH1, 29% for OTPH2.

**Scheme 5:**
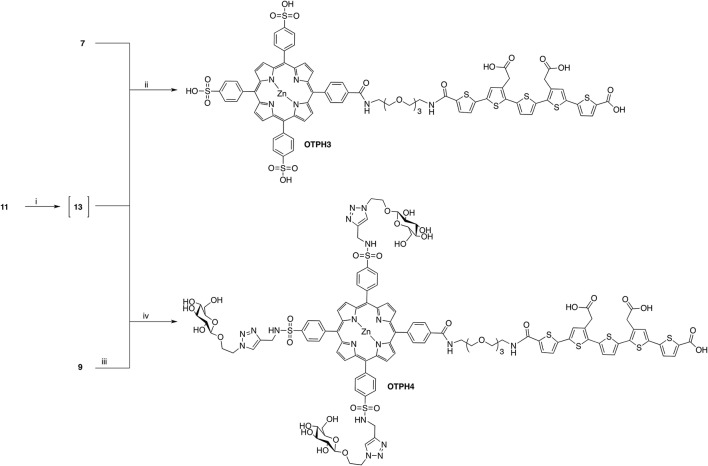
Reagents and conditions: (i) DCM:TFA, 4:1, r.t. (ii) 1. HATU, DIPEA, DMF, r.t. 2. NaOH, H_2_O, THF, r.t. 3. Zn(OAc)_2_(H_2_O)_2_, r.t. 78% (iii) NHS, EDC, DMF, r.t. (iv) 1. DIPEA, DMF, r.t. 2. NaOH, H_2_O, THF, r.t. 19%.

Amide bond formation was used for tethering LCO-linker conjugates **11** and **12** to porphyrin moieties **5**, **7**, and **9** (Schemes 4, 5). The N-Boc protected amine of the linkers was deprotected in the solution of DCM:TFA, 4:1, at room temperature and the corresponding amines were used in the coupling to the porphyrinic acids. For syntheses of target compounds **OTPH1** and **OTPH2**, NHS-activated porphyrin **5** was used together with LCOs **13** and **14**, respectively. The resulting compounds were directly deprotected at the methyl esters yielding **OTPH1** and **OTPH2** (Scheme 4) in good to satisfactory yields. The close proximity of the oligothiophene and the porphyrin moiety in **OTPH2**, as compared to **OTPH1**, gave rise to the tendency of aggregation and poor solubility, both as the methyl ester in organic solvents and as the carboxylic acid in aqueous solutions, which seriously complicated purification and characterization.

In the synthesis of compound **OTPH3**, porphyrin **7** was reacted with oligothiophene **13** in a HATU-mediated amine bond formation reaction, followed by hydrolysis of the methyl esters on the LCO (Scheme 5). **OTPH3** was isolated in a good yield using preparative HPLC. In order to obtain **OTPH4**, porphyrin **9** was NHS-activated *in situ* and let react with oligothiophene **13** in the presence of TEA. Final deprotection gave the desired glycoconjugated compound (Scheme 5).

All five target OTPHs were characterized by ^1^H- and ^13^C-NMR in deuterated DMSO in their protonated carboxylic acid form if any present. The NMR-analyses together with mass- and IR spectra confirmed the identity of the synthesized OTPHs. For the photophysical and histological studies the OTPHs were turned into the corresponding sodium carboxylates that were used in 1.5 mM stock solutions in de-ionized water or de-ionized water with 10% DMSO (**OPTH2**).

### Optical characterization of the OTPHs

OTPHs dissolved in PBS buffer (pH 7.4) showed characteristic porphyrin absorption with the Soret band around 420 nm for **OTPH1** and **OTPH2** and around 430 nm for **OTPH3** and **OTPH4** (Figure 1B). The two Q-bands expected from metallated porphyrins appeared at around 550 nm and 580 nm for **OTPH1** and **OTPH2**, whereas **OTPH3** and **OTPH4** displayed these bands at around 560 nm and 610 nm (Figure 1C). The red-shifted absorption of **OTPH3** and **OTPH4** compared to **OTPH1** and **OTPH2** can be ascribed to the two extra phenylic meso-substituents at the porphyrin core. In contrast to the OTPHs, the oligothiophene, p-FTAA, without any porphyrin moiety displayed no Q-bands (Figure 1C), as well as a broader absorption between 300 and 500 nm (Figure 1B). Upon excitation at 420 nm p-FTAA displayed an emission spectrum having a maximum around 550 nm (Figure 1D). For the OTPHs, the porphyrin contribution of **OTPH1** and **OTPH2** showed blue-shifted emission, with peaks at around 600 and 650 nm, as compared to **OTPH3** and **OTPH4**, with peaks at around 620 and 670 nm (Figure 1D). Interestingly, the expected emission of p-FTAA at 550 nm was strongly decreased for **OTPH1** and **OTPH2** and completely absent for **OTPH3** and **OTPH4**, suggesting that the emission from the oligothiophene moiety was sufficiently quenched by the porphyrin moiety.

### Emission characteristics of OTPHs bound to Aβ aggregates in tissue

**Figure 2:**
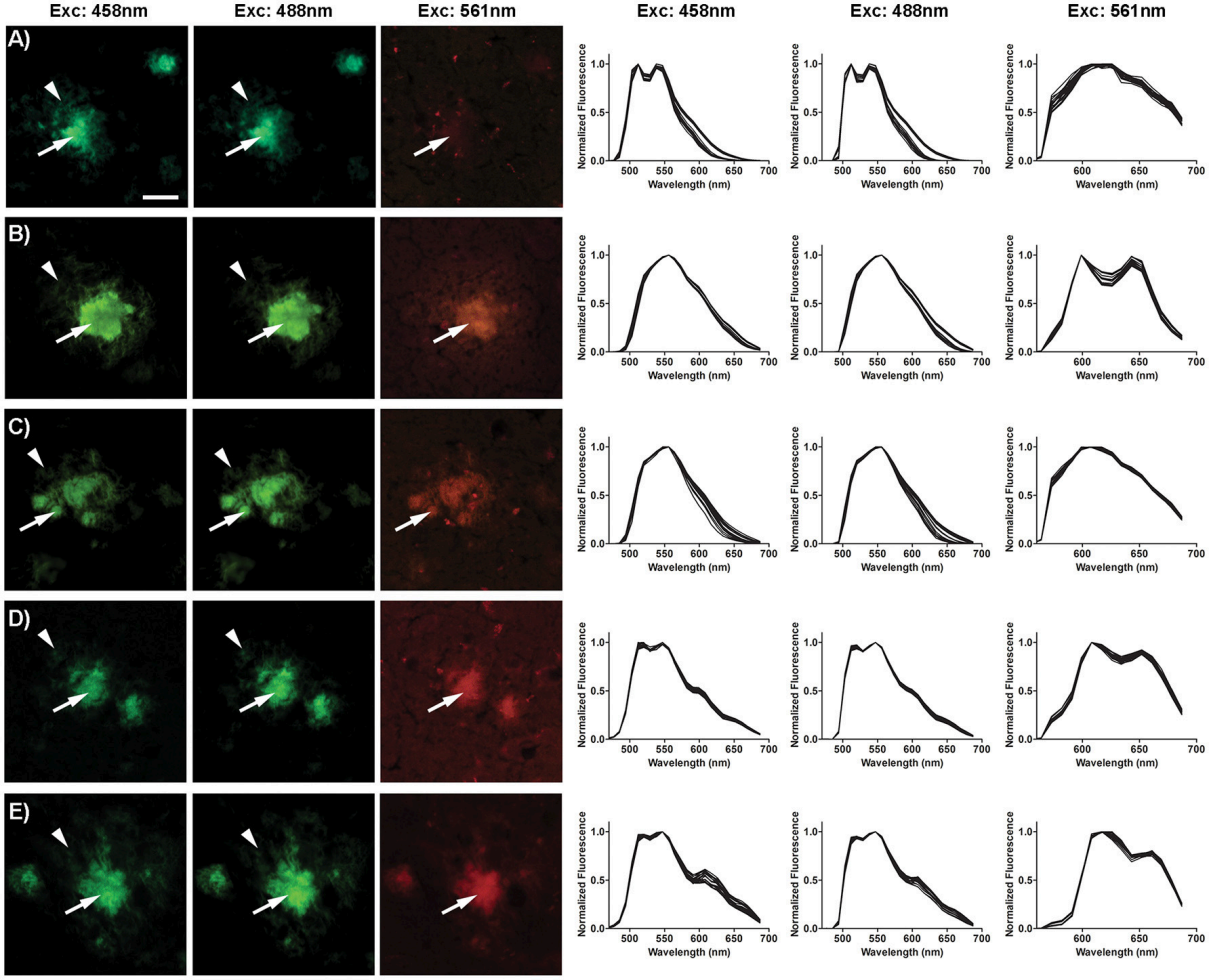
Optical characterization of OTPH stained Aβ deposits in brain tissue section from APPPS1 mice. Fluorescence images (left) and emission spectra (right) of Aβ deposits stained with p-FTAA **(A)**, OTPH1 **(B)**, OTPH2 **(C)**, OTPH3 **(D)**, and OTPH4 **(E)**. Three different exaction wavelengths (458, 488, and 561 nm) were used for all molecules. The Aβ deposits are composed of compact cores (white arrows) and a diffuse exterior (white arrow heads). Emission spectra were collect from 20 to 30 individual Aβ deposits. Scale bar represents 20 μm.

In the same fashion as in the previously reported studies on LCO-based molecules (Åslund et al., 2009; Arja et al., 2013; Klingstedt et al., 2015), we next applied OTPHs for histological staining of brain tissue sections from transgenic APPPS1 mice with Aβ pathology. Aβ aggregates could easily be detected due to bright fluorescence signal from the bound dyes (Figure 2), confirming that tethering bulky porphyrins to p-FTAA does not significantly influence the dyes selectivity and specificity for protein deposits in tissue sections. To investigate, the molecular interplay between the two optical moieties, fluorescence images and emission spectra from Aβ deposits stained with p-FTAA or the respective OTPH were recorded with excitation at 458, 488, or 561 nm (Figure 2). At 458 nm both optically active parts of the molecules are excited to some extent, while at 488 nm mainly p-FTAA is excited and at 561 nm predominantly the porphyrin moieties are excited. For **OTPH1** and **OTPH2**, excitation at 458 nm and 488 nm generated similar emission spectra as obtained for p-FTAA (Figures 2A–C, right panel), indicating that the p-FTAA emission was dominant while the emission peaks from the porphyrin moieties were absent. However, the characteristic double peaks observed from Aβ-bound p-FTAA (Figure 2A, right panel) were lacking and this phenomenon have also been observed for an azide functionalized p-FTAA, having an azide moiety linked to the thiophene backbone via a tetra ethylene glycol spacer (Johansson et al., 2015). Nevertheless, in contrast to the emission spectra of **OTPH1** and **OTPH2** in PBS (Figure 1D) where the fluorescence of p-FTAA was significantly quenched, the emission from the oligothiophene moiety was dominant for **OTPH1** and **OTPH2** bound to Aβ deposits, suggesting that these OTPHs adopt different stereospacial conformation upon binding to the protein aggregates. A similar spectral transition was also observed for **OTPH1** when mixed with recombinant Aβ1-42 fibrils (Arja et al., 2013), indicating that the molecular interplay between the two optical moieties are altered upon interaction with protein aggregates. **OTPH3** and **OTPH4** bound to Aβ-aggregates generated considerably different spectral signature upon excitation at 458 nm and 488 nm (Figures 2D,E, right panel). Similarly, to **OTPH1** and **OTPH2**, the p-FTAA emission of these dyes was observed when bound to protein deposits. However, in contrast to **OTPH1** and **OTPH2**, the well-defined emission maxima at 515 and 545 nm, characteristic for p-FTAA bound to protein aggregates, was evident. In addition, the emission from the porphyrin moiety could be observed as shoulders at 620 and 670 nm (Figures 2D,E, right panel). Hence, the molecular interplay between the oligothiophene and the porphyrin moieties were considerably different for **OTPH3** and **OTPH4** compared to **OTPH1**, verifying that the chemical composition of the porphyrin moiety is influencing the interplay between the two optical moieties.

Upon excitation at 561 nm, p-FTAA stained deposits showed only weak fluorescence (Figure 2). In contrast, all the OTPHs displayed bright staining of the core of the Aβ aggregates, but emission from the diffuse exterior of the deposits was lacking (Figure 2). Thus, in a similar fashion as observed previously (Arja et al., 2013), the emission from the porphyrin moiety could only be observed from certain areas of the aggregates. Earlier studies (Nilsson et al., 2007; Nyström et al., 2013) have shown morphological differences between the core and the diffuse exterior of the aggregates and these structural alterations might force the OTPHs to bind in different configurations that will lead to distinct spectroscopic signatures from the OTPHs. Interestingly, **OTPH1**, **OTPH3**, and **OTPH4** displayed a characteristic porphyrin double peak emission, whereas the **OTHP2** showed a less defined spectrum with broader emission, suggesting that the length of the spacer between the oligothiophene and the porphyrin are influencing the molecular interplay between the two optical moieties. In addition, the emission from **OTPH4** bound to Aβ deposits was much stronger compared to the other OTPHs, indicating that glycoconjugated porphyrin moiety is influencing the optical performance of the molecule.

### Fluorescence life time imaging of OTPHs bound to Aβ aggregates

**Figure 3:**
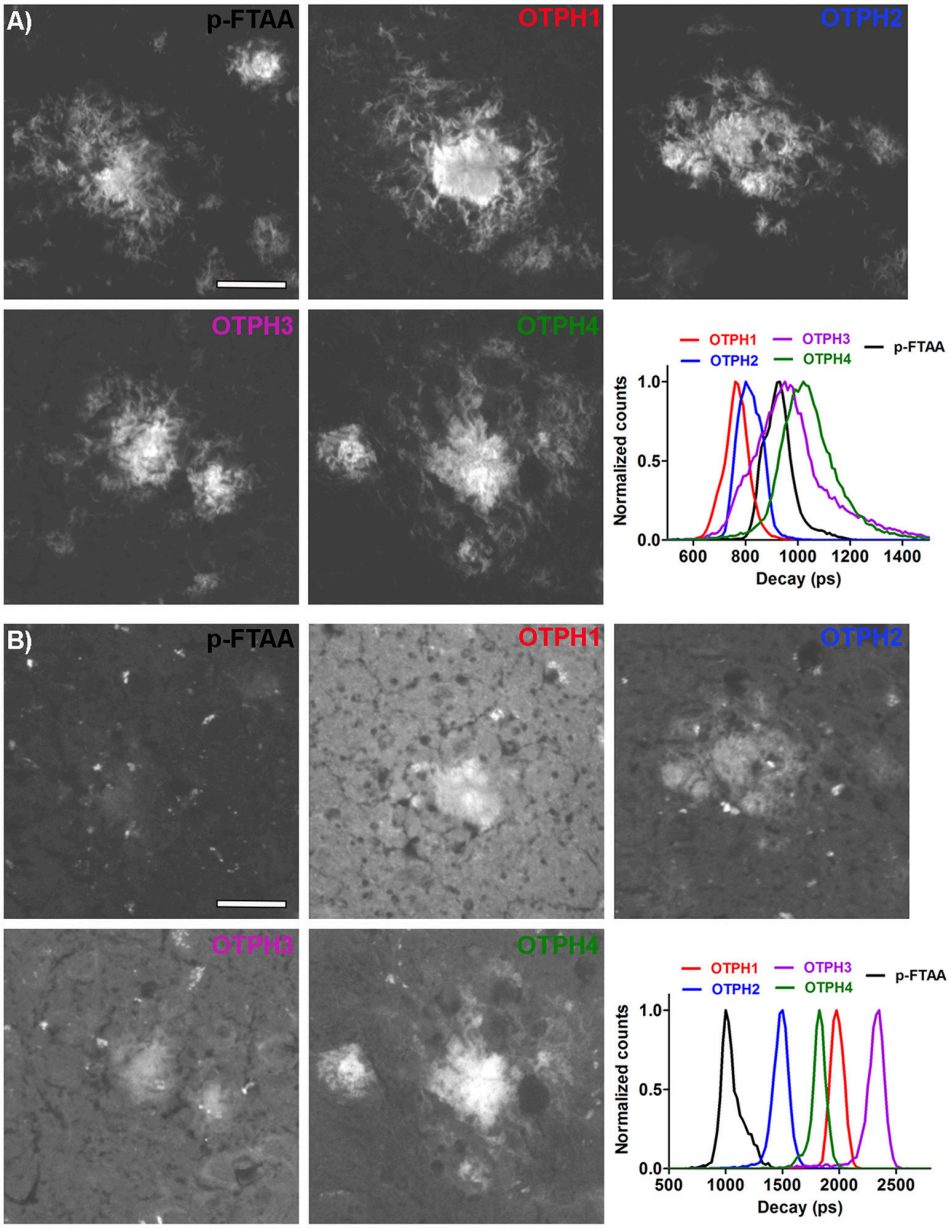
Fluorescence life time images and intensity-weighted mean lifetime (t_i_) distributions of p-FTAA and OTPHs stained Aβ deposits in brain tissue section from transgenic mice with AD pathology. **(A)** Excitation at 490 nm. **(B)** Excitation at 561 nm. Decays were collect from 20 to 30 individual Aβ deposits. Scale bar represents 20 μm.

To investigate the fluorescence characteristics of the OTPHs in more detail, we applied FLIM of OTPH-stained Aβ deposits (Figure 3). FLIM experiments were carried out with excitation at 490 nm and 565 nm, respectively, and the acquired decay curves were fitted with a bi-exponential decay function and two components of the fit. When excited at 490 nm, p-FTAA displayed intensity-weighted mean lifetime (t_i_) distributions between 800 and 1,000 ps and both the core as well as the diffuse exterior of the aggregates could be visualized (Figure 3A). Likewise, the core and the diffuse exterior of the Aβ deposits could also be observed with all the OTPHs, but the mean lifetime distributions for all the OTPHs was strikingly different compared to p-FTAA. **OTPH2** displayed decays between 700 and 950 ps, whereas **OTPH1** showed decays between 600 to 900 ps, showing that the spacing between the oligothiophene and the porphyrin moiety slightly influenced the decay. In addition, **OTPH3** and **OTPH4** demonstrated broader decays between 800–1,400 and 700–1,400 ps, respectively. The longer decays observed for **OTPH3** and **OTPH4** is most likely associated with the porphyrin moiety, suggesting that both the optical active groups, the oligothiophene and the porphyrin, are contributing to the life time distribution. This observation is also in agreement with the emission characteristics from **OTPH3** and **OTPH4** bound Aβ deposits (Figures 2D,E, right panel), since two minor shoulders at 620 and 670 nm were observed in the emission spectra when using a similar excitation wavelength (488 nm).

Upon excitation at 565 nm, decays could only be observed from the parts of the deposits displaying the characteristic porphyrin emission (Figure 3B). **OTPH1** and **OTPH4** displayed similar intensity-weighted mean lifetime (t_i_) distributions ranging from 1,700 to 2,200 ps, whereas **OTPH2** and **OTPH3** displayed shorter (1,400–1,600 ps) and longer decays (2,200–2,500 ps), respectively. In contrast, p-FTAA displayed very weak staining and decays between 900 and 1,200 ps. Thus, the longer decays observed from the OTPHs are related to the porphyrin moiety. Similar to the observation from the emission characteristics, **OTPH4** displayed the most intense staining of the Aβ deposits (Figure 3B).

### Spectral assessment and fluorescence life time imaging of OTPHs bound to distinct aggregated Aβ morphotypes

**Figure 4:**
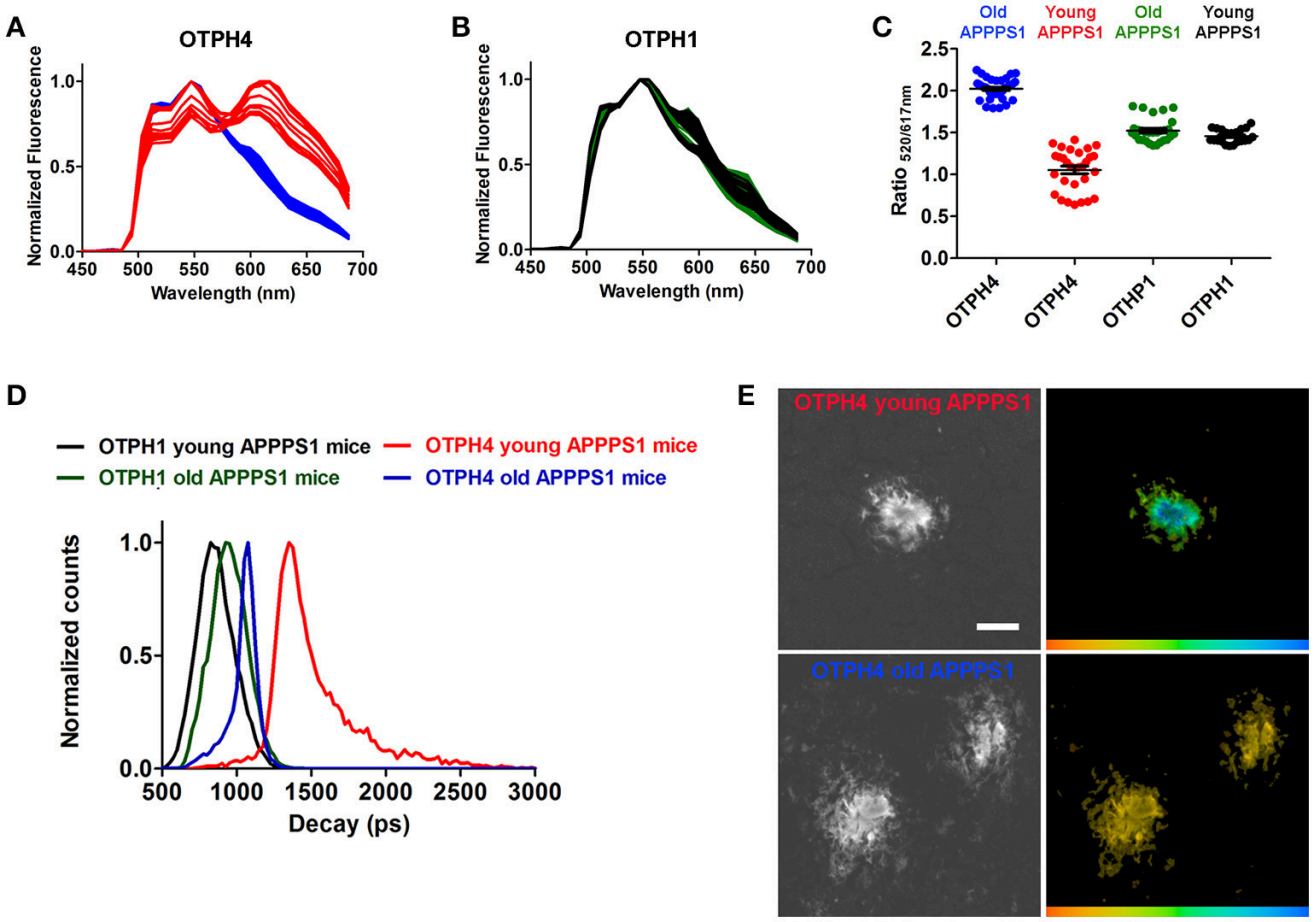
Spectral assessment and fluorescence life time imaging of OTPH1 and OTPH4 stained Aβ deposits in brain tissue section from a young (148 days) or an old (533 days) APPPS1 transgenic mouse with AD pathology. **(A)** Emission spectra from OTPH4 bound to Aβ deposits in brain tissue section from young (red) or old (blue) APPPS1 transgenic mice. **(B)** Emission spectra from OTPH1 bound to Aβ deposits in brain tissue section from young (black) or old (green) APPPS1 transgenic mice. **(C)** Diagram showing the ratio of intensity of the emitted light at 520 and 617 nm, Ratio_520/617nm_, for OTPH1 or OTPH4 stained Aβ deposits in brain tissue section from young or old APPPS1 transgenic mice. The fluorescence intensity at 520 nm reflects oligothiophene (p-FTAA) emission, whereas the intensity at 617 nm reflects porphyrin emission. Three to five spectra from the center of 5–10 randomly selected plaques from each mouse were subjected to ratio-metric calculation. Error bars represent SEM. **(D)** Intensity-weighted mean lifetime (t_i_) distributions of OTPH1 and OTPH4 bound to Aβ deposits in brain tissue section from young or old APPPS1 transgenic mice. Decays were collect from 10 to 20 individual Aβ deposits for each mouse. **(E)** Fluorescence lifetime images of OTPH4 stained Aβ deposits in brain tissue section from young or old APPPS1 transgenic mice. The color bar represents lifetimes from 500 ps (orange) to 3,000 ps (blue) and the images are color coded according to the representative lifetime. The fluorescence lifetimes were collected with excitation at 490 nm. Scale bar represents 20 μm.

Recent studies (Cohen et al., 2015; Rasmussen et al., 2017; Condello et al., 2018) have shown that multiple aggregate morphotypes of Aβ can be found human AD brain. Likewise, in transgenic mouse models, conformational rearrangement within Aβ deposits have been shown to be an age-dependent phenomenon (Nyström et al., 2013; Klingstedt et al., 2015), suggesting that different aggregate species of Aβ are present during different stages of the pathological process. Therefore, we next tested **OTPH1** and **OTPH4** on brain tissue sections from young or old transgenic APPPS1 mice with Aβ pathology. This pair of OTPHs was selected to examine if an OTPH with a glycoconjugated porphyrin moiety, **OTPH4**, would improve the optical assessment of heterogeneous Aβ deposits compared to the previously reported **OTPH1** (Arja et al., 2013). As shown in Figure 4A, **OTPH4** bound to Aβ deposits in old mice displayed similar emission spectra, with a dominant contribution from the oligothiophene moiety, as described above (Figure 2E). In contrast, when bound to Aβ deposits in young mice, spectra displaying an even distribution of emission characteristic from the oligothiophene, 520 and 545 nm, as well as the porphyrin moiety, 617 and 670 nm, could be observed (Figure 4A). Hence, as reflected in the emission spectra, **OTPH4** binds in completely different fashions when bound to aggregate Aβ morphotypes in young or old APPPS1 mice. The high contribution of the porphyrin emission observed from **OTPH4** bound to aggregate Aβ morphotypes in young mice might be associated with Förster resonance energy transfer (FRET) from the oligothiophene to the porphyrin, since the porphyrin display low absorption at 488 nm (Figure 1). Alternatively, the interaction with these Aβ deposits favors a configuration of the porphyrin that displays more efficient emission. Interestingly, Aβ morphotypes in young or old mice showed similar spectral signatures when stained with **OTPH1** (Figure 4B). When plotting the ratio of the intensity of the emission from the oligothiophene and the porphyrin moiety, Ratio_520/617nm_, differentiation of Aβ morphotypes in young or old mice could be afforded by **OTPH4** but this variation was not observed for **OTPH1** (Figure 4C). Thus, spectral separation of the aggregate Aβ morphotypes could only be afforded by **OTPH4**, suggesting that the glycoporphyrin moiety might be a chemical determinant for achieving OTPHs that can be utilized for enhanced optical assessment of heterogeneous Aβ deposits.

The **OTPH4** specific differentiation of aggregated Aβ morphotypes could also be confirmed by FLIM. When excited at 490 nm, **OTPH4** bound to Aβ deposits in old mice displayed intensity-weighted mean lifetime (t_i_) distributions between 800 and 1,200 ps, whereas distributions of longer decays, 1,200–2,500 ps, were achieved from **OTPH4** stained Aβ deposits in young mice (Figure 4D). These longer decays are presumably associated with the porphyrin moiety and this finding confirmed the observation of the high contribution from the porphyrin in the emission spectra (Figure 4A). The significant difference between Aβ morphotypes in young or old mice could also be visualized by color-coded fluorescence lifetime images (Figure 4E). In agreement with the spectral assessment, **OTPH1** showed similar intensity-weighted mean lifetime (t_i_) distributions when bound to Aβ deposits in young and old mice (Figure 4D), verifying that **OTPH4** was superior compared to **OTPH1** in distinguishing aggregated Aβ morphotypes.

## Conclusions

In conclusion, a series of OTPHs was synthesized and characterized toward aggregated Aβ morphotypes. Fluorescence studies revealed that the chemical identity of the porphyrin moiety, as well as the spacing between the two optically active moieties, influenced the OTPHs performance for fluorescent assignment of Aβ deposits. In addition, an OTPH functionalized with a glucose-conjugated porphyrin moiety was identified as a superior dye for optical assignment of age-dependent Aβ morphotypes. Our findings will aid in the chemical design of novel OTPHs and we foresee that OTPHs will be vital molecular tools to unravel the roles of aggregated proteinaceous morphotypes in proteopathic neurodegenerative diseases.

## Author contributions

KPRN designed and performed research, analyzed data, and wrote the manuscript. KA and ME synthesized molecules, performed research, analyzed data, and wrote the manuscript.

### Conflict of interest statement

The authors declare that the research was conducted in the absence of any commercial or financial relationships that could be construed as a potential conflict of interest.
