# Supplementary material for: Synthesis and Characterization of Oligothiophene–Porphyrin-Based Molecules That Can Be Utilized for Optical Assignment of Aggregated Amyloid-β Morphotypes
Source: Front Chem. 2018 Sep 3;6:391. doi: 10.3389/fchem.2018.00391 (PMC6129614; doi:10.3389/fchem.2018.00391)
Supplement: Supplementary file 1 [file Data_Sheet_1.PDF]

Supplementary Material

Synthesis and characterization of oligothiophene–porphyrin-based ligands that can be utilized for optical assignment of aggregated amyloid-β morphotypes

Katriann Arja[1], Mathias Elgland[1], and K. Peter R. Nilsson[1]*

[1]Division of Chemistry, Department of Physics, Chemistry and Biology, Linköping University, Linköping, Sweden

*** Correspondence:** K. Peter R. Nilsson, E-mail: peter.r.nilsson@liu.se

1 Supplementary Methods and schemes

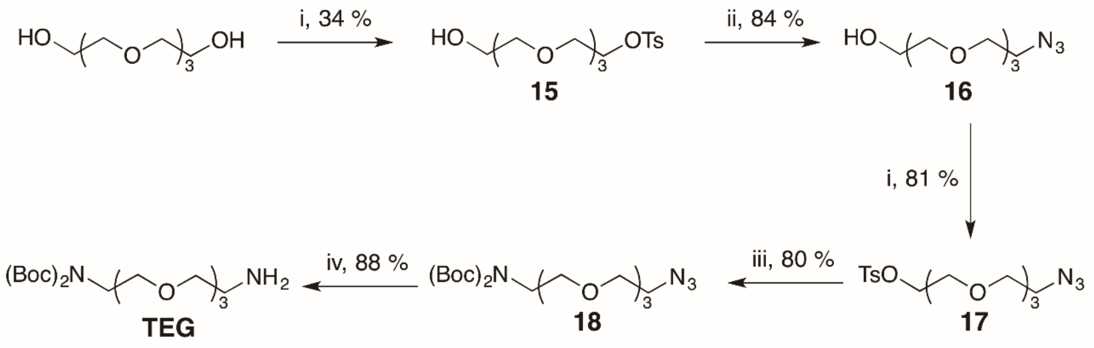

Scheme S1. Synthesis of the TEG linker. General conditions and reagents: i) TsCl, Py, 0 °C to r.t.; ii) NaN_3, DMF, 60 °C; iii) $NH(Boc)_2$, K_2CO_3, THF, 70 °C; iv) 1. PPh_3, THF, 60 °C; 2. H_2O.

Compound 15

Tetraethylene glycol (10.0 g, 51.5 mmol) was suspended in pyridine (60 mL) at 0 °C. Tosyl chloride (9.83 g, 51.5 mmol), suspended in pyridine (60 mL), was added dropwise. The reaction vessel was lifted from the ice bath and the mixture was stirred at room temperature for 3 hours. Pyridine was removed via co-evaporation with xylene by rotary evaporator and the yellowish oil obtained purified by silica gel chromatography eluting with ethyl acetate. The yield gained of the desired product was 6.13 g (17.6 mmol, 34 %). NMR $CDCl_3$ (300 MHz): ^1H: δ (ppm) 2.45 (s, 3H), 3.59-3.71 (m, 14H),

4.17 (t, 2H, J = 4.70 Hz), 7.33 (d, 2H, J = 8.22 Hz), 7.80 (d, 2H, J = 8.22 Hz). ^{13}C: δ (ppm) 61.7, 68.7, 69.2, 70.3, 70.5, 70.6, 70.7, 72.5, 128.0, 129.8, 133.1, 144.8.

Compound 16

11 (6.12 g, 17.6 mmol) and sodium azide (1.37 g, 21.1 mmol) were dissolved in DMF and the mixture was allowed to stir at 60 °C for 2 hours. The solvent was co-evaporated with xylen and the sodium tosylate was partly removed on filter. The crude product was purified on silica gel eluting with ethyl acetate. 3.23 g oilish product was obtained in 84 % yield. NMR $CDCl_3$ (300 MHz): ^{1}H: δ (ppm) 2.78 (t, 1H, J = 5.87 Hz), 3.39 (t, 2H, J = 5.28 Hz), 3.59-3.62 (m, 2H), 3.66-3.76 (m, 14 H). ^{13}C: δ (ppm) 50.7 (CH_2), 61.7, 70.0, 70.3, 70.6, 70.66, 70.69, 72.5.

Compound 17

12 (3.23 g, 14.7 mmol) was dissolved in pyridine (14 mL) and cooled to 0 °C. Tosyl chloride, dissolved in pyridine (17 mL), was added dropwise and the reaction mixture was allowed to stir for 2.5 hours while the temperature slowly reached room temperature. Under continuous TLC-checks two new portions of tosyl chloride (2x1.403 g, 7.357 mmol) were added within an hour and the reaction was declared complete after the total of 5 hours. The mixture was washed in toluene (50 mL) with 1 M HCl (4x40 mL), the solvent reduced via vacuum and the crude purified by FC on silica. The mobile phases used were toluene:ethyl acetate, 6:1, followed by toluene:ethyl acetate, 1:1. The product was gained as a slightly yellow oil in 81 % yield (4.43 g). NMR $CDCl_3$ (300 MHz): ^{1}H: δ (ppm) 2.45 (s, 3H), 3.35 (t, 2 H, J = 5.00 Hz), 3.50-3.70 (m, 12 H), 4.15 (t, 2 H, J = 4.69 Hz), 7.35 (d, 2 H, J = 8.80 Hz), 7.79 (d, 2H, J = 8.22 Hz). ^{13}C: δ (ppm) 21.4, 50.5, 68.4, 69.2, 69.8, 70.3, 70.38, 70.41, 70.5, 127.7, 129.7, 132.8, 144.7.

Compound 18

13 (4.43 g, 11.9 mmol), potassium carbonate (3.69 g, 11.9 mmol) and *N,N*-diBoc-ammonia (3.09 g, 14.2 mmol) were dissolved in THF (35 mL) and the mixture was heated to 70 °C. The reaction was allowed to stir for 20 hours. The reaction mixture was washed in diethyl ether (50 mL) with water (100 mL). Co-evaporation with xylene on rotary evaporator gave a crude oil, which was purified on silica gel column. Mobile phases used were toluene: ethyl acetate, 18:1, followed by 6:1 and finally 2:1. The product was obtained as a pale-yellow oil in 80 % yield (3.99 g). NMR $CDCl_3$ (300 MHz): ^{1}H: δ (ppm) 1.47 (s, 18 H), 3.35 (t, 2 H, J = 5.29 Hz), 3.55-3.70 (m, 12 H), 3.75 (t, 2 H, J = 7.04 Hz). ^{13}C: δ (ppm) 28.0, 45.2, 50.7, 69.3, 70.0, 70.2, 70.6, 70.7, 82.2, 152.6.

TEG

13 (3.99 g, 9.53 mmol), triphenylphosphine (3.00 g, 11.4 mmol) and 2 drops of water were dissolved in THF (35 mL). The mixture was heated to 60 °C and allowed to stir for 4 hours, whereupon another small portion of water (1 mL) was added. The product had difficulty in coming over to a slightly acidic water phase while washing it in water (50 mL, pH ≅ 5) with DCM (4x30 mL). Therefore, all the product was moved back to organic phase when washing by now basic aqueous phase (Na_2CO_3(aq), pH ≅ 12) with DCM (4x30 mL). The solvent was reduced *in vacuo* and the crude purified by FC on silica eluting with the following mobile phases: ethyl acetate, then DCM:methanol, 5:1, with 2 % TEA, and finally DCM:methanol, 4:1, with 2 % TEA. The product was obtained as a slightly yellow oil in 88 % yield (3.26 g). NMR $CDCl_3$ (300 MHz): ^{1}H: δ (ppm) 1.47 (s, 18 H), 1.70 (s, 2 H), 2.84 (t, 2H, *J* = 5.29 Hz, 4.70 Hz), 3.49 (t, 2H, *J* = 5.29 Hz), 3.55-3.65 (m, 10 H), 3.76 (t, 2H, *J* =6.47 Hz, 5.87 Hz). ^{13}C: δ (ppm) 28.0, 41.7, 45.2, 69.3, 70.2, 70.3, 70.6, 73.3, 82.2, 152.6.

2 Supplementary NMR and IR spectra

OTPH1

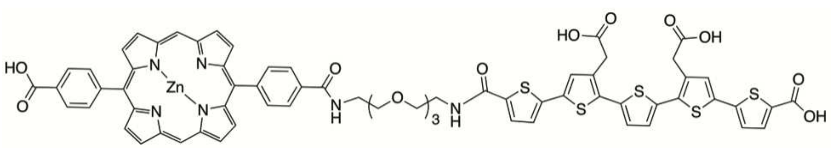

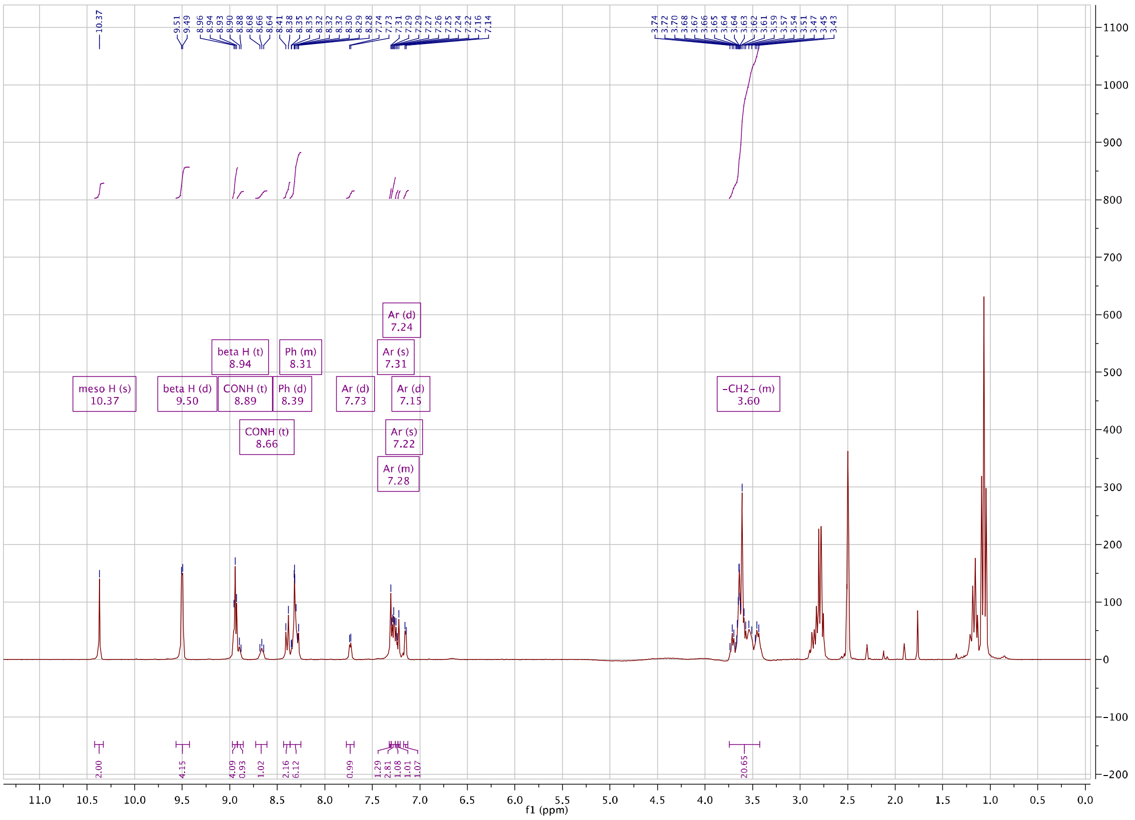

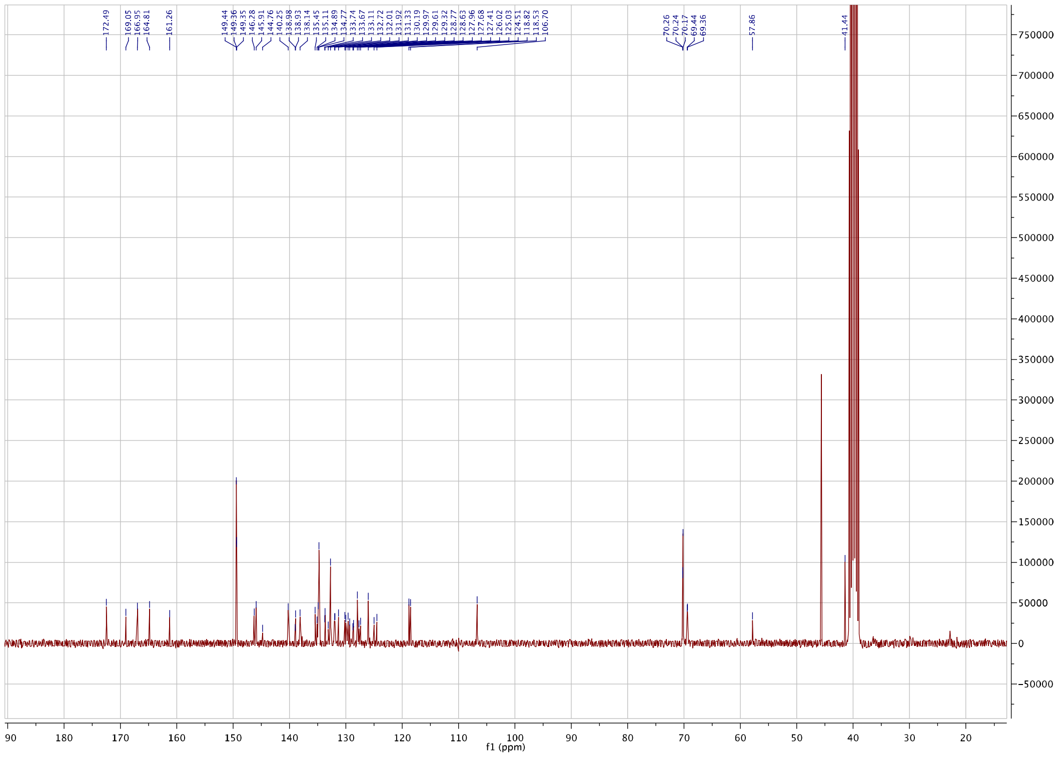
f1 (ppm)

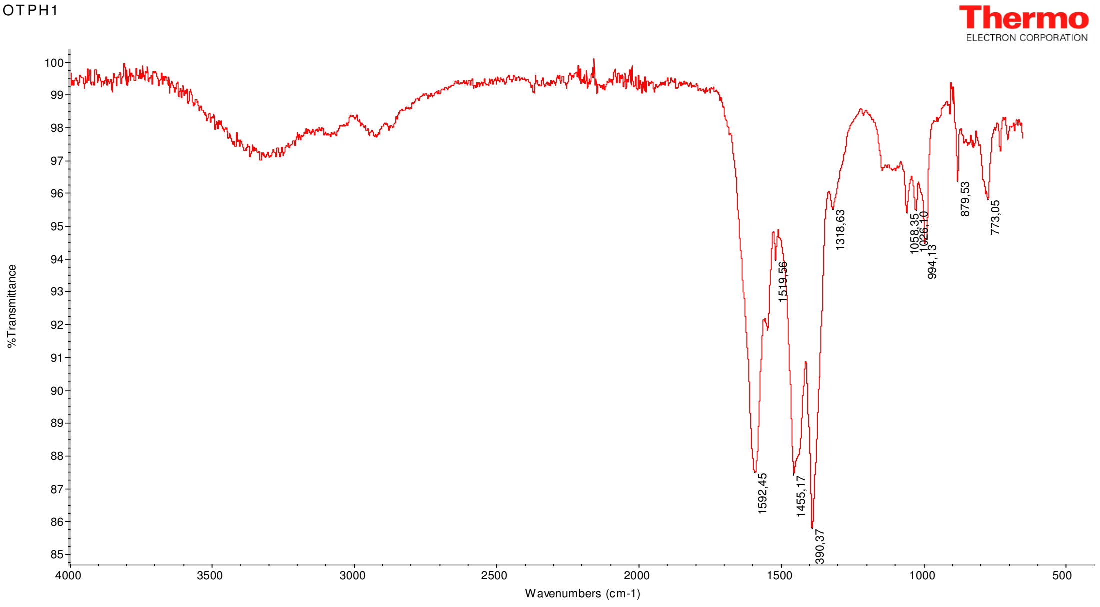
OTPH1
Thermo
ELECTRON CORPORATION
%Transmittance
Wavenumbers (cm-1)
1592,45
1519,56
1455,17
390,37
1318,63
1058,35
1026,10
994,13
879,53
773,05
Wed Apr 11 15:38:32 2018 (GMT+02:00)

OTPH2

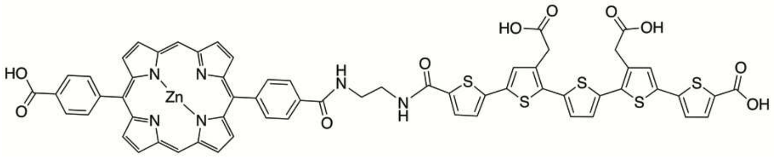

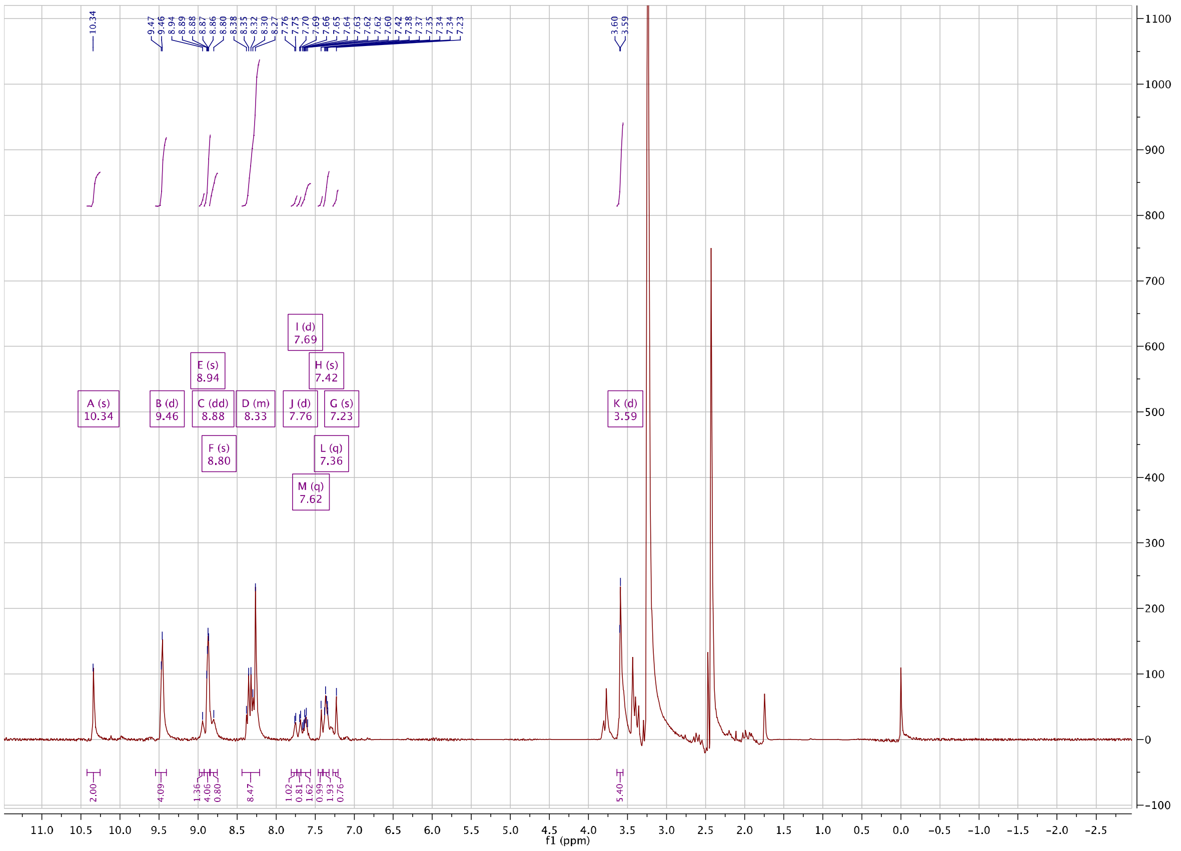

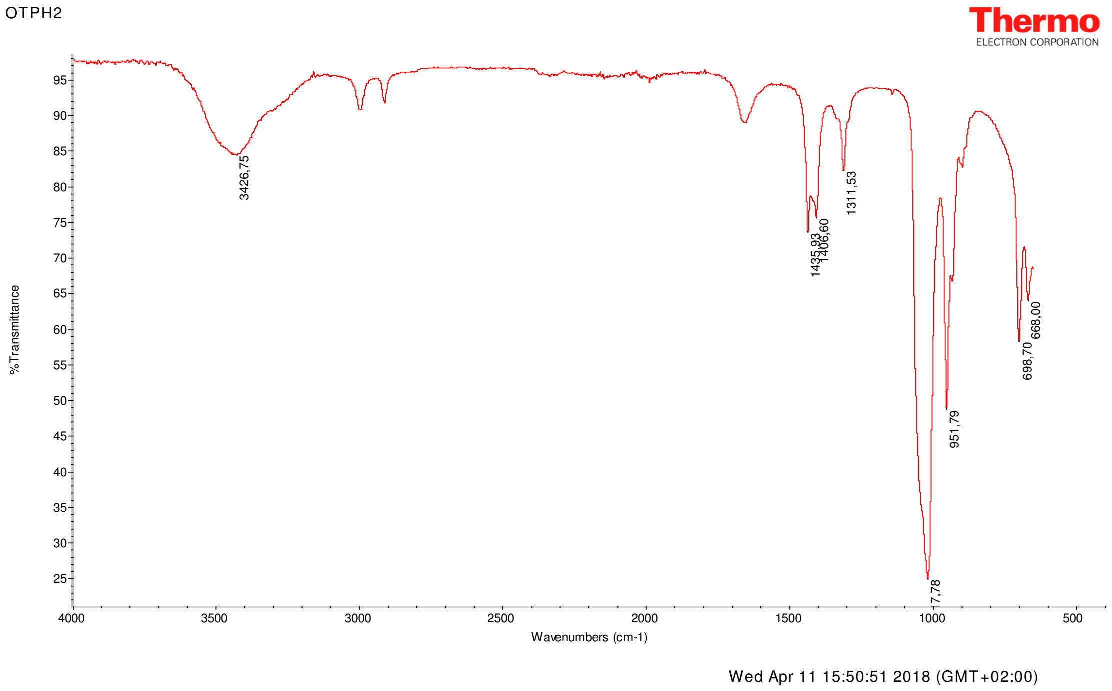
OTPH2
Thermo
ELECTRON CORPORATION
%Transmittance
95
90
85
80
75
70
65
60
55
50
45
40
35
30
25
3426,75
1435,93
1406,60
1311,53
17,78
951,79
698,70
668,00
4000
3500
3000
2500
2000
1500
1000
500
Wavenumbers (cm-1)
Wed Apr 11 15:50:51 2018 (GMT+02:00)

OTPH3

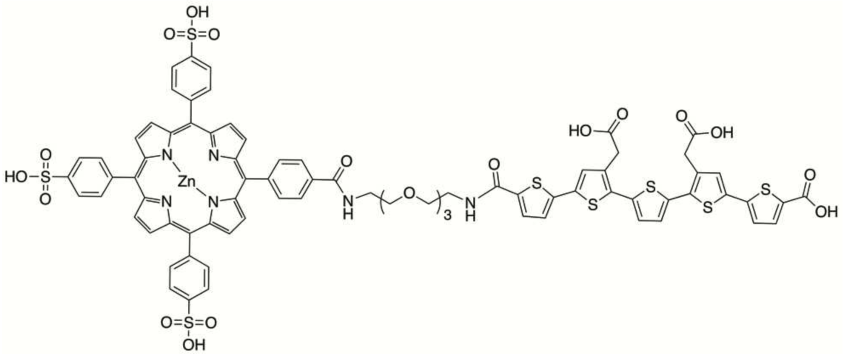

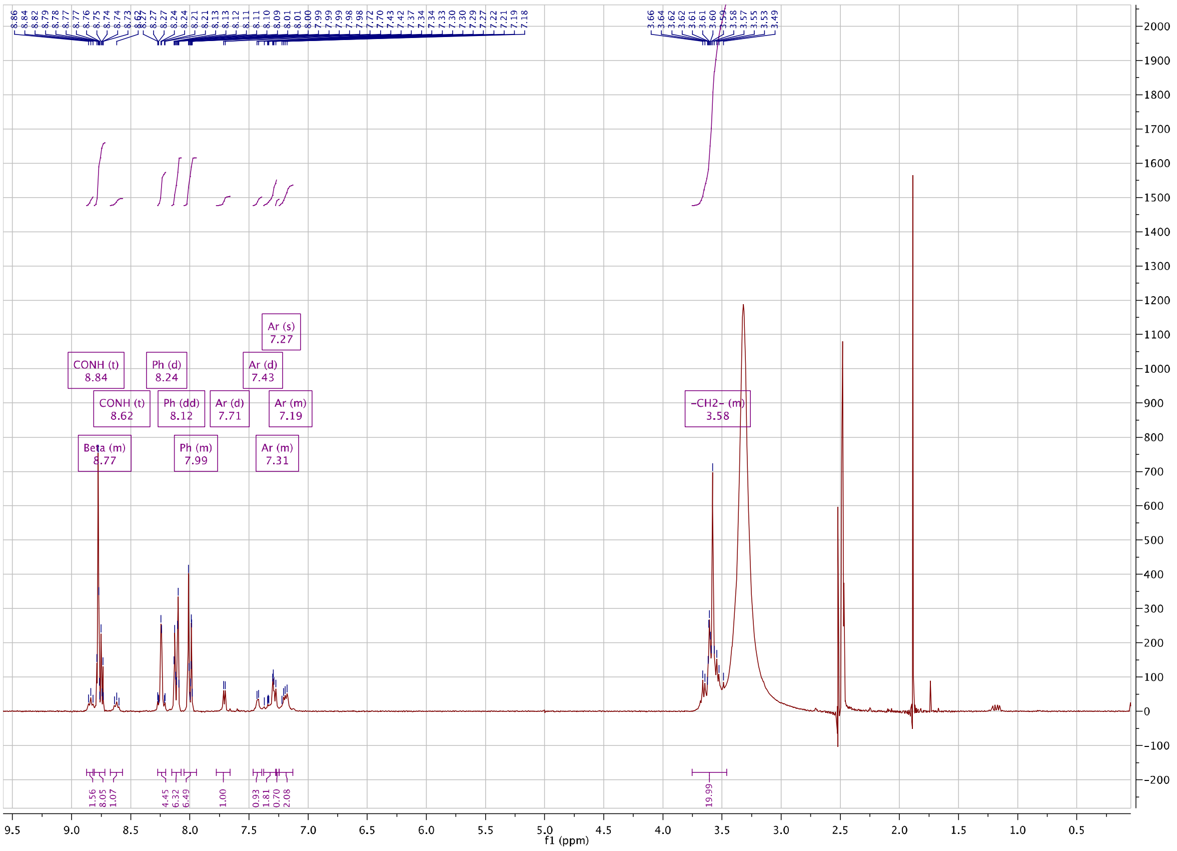
CONH (t)
8.84
CONH (t)
8.62
Beta (m)
8.77
Ph (d)
8.24
Ph (dd)
8.12
Ph (m)
7.99
Ar (d)
7.71
Ar (d)
7.43
Ar (s)
7.27
Ar (m)
7.19
Ar (m)
7.31
-CH2- (m)
3.58
f1 (ppm)

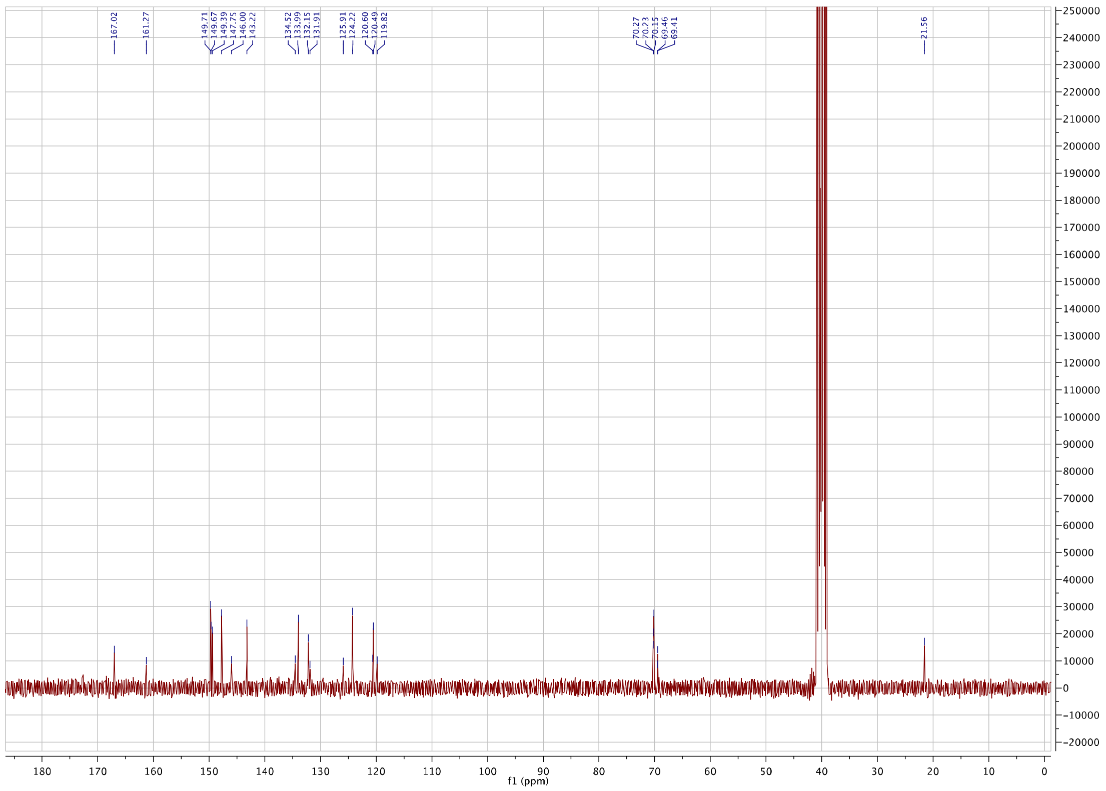
167.02
161.27
149.71
149.67
149.39
147.75
146.00
143.22
134.52
133.99
132.15
131.91
125.91
124.22
120.60
120.49
119.82
70.27
70.23
70.15
69.46
69.41
21.56
f1 (ppm)

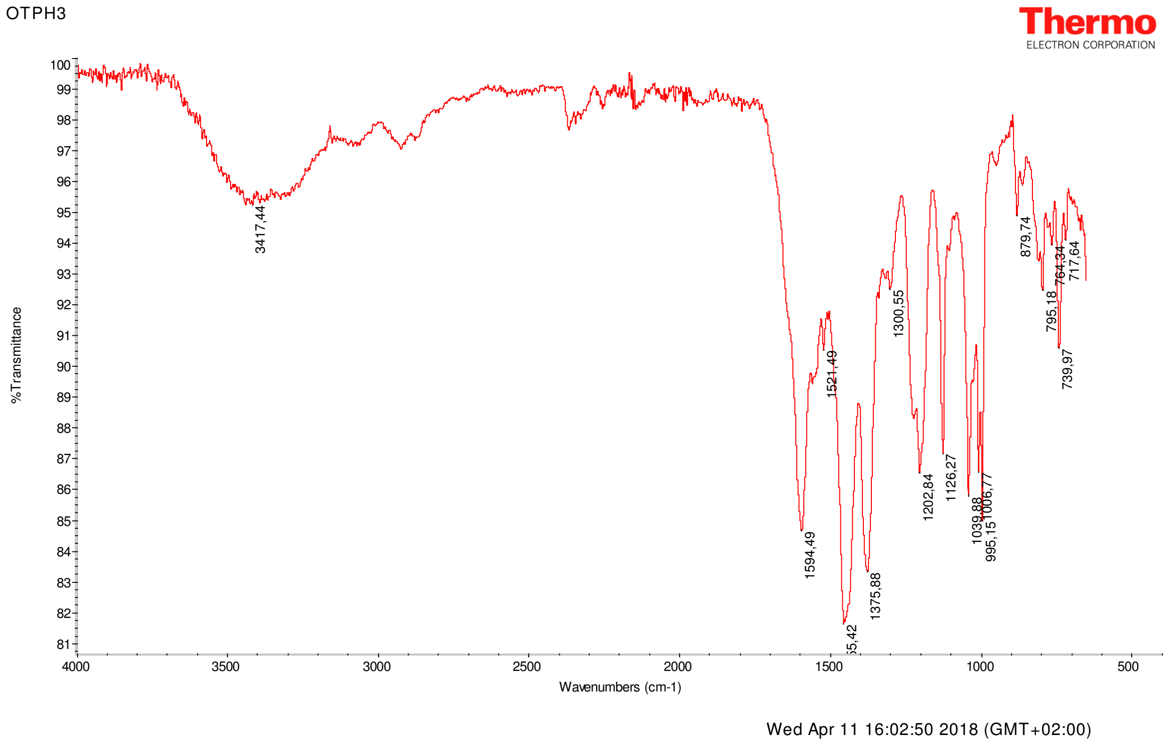
OTPH3
Thermo
ELECTRON CORPORATION
%Transmittance
Wavenumbers (cm-1)
3417,44
1594,49
1521,49
5,42
1375,88
1300,55
1202,84
1126,27
1039,88
995,15
1006,77
879,74
795,18
764,34
739,97
717,64
Wed Apr 11 16:02:50 2018 (GMT+02:00)

OTPH4

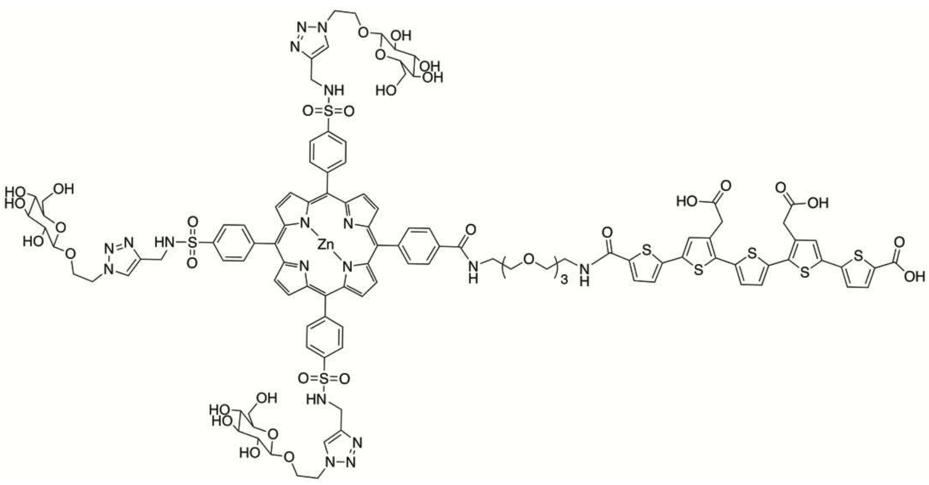

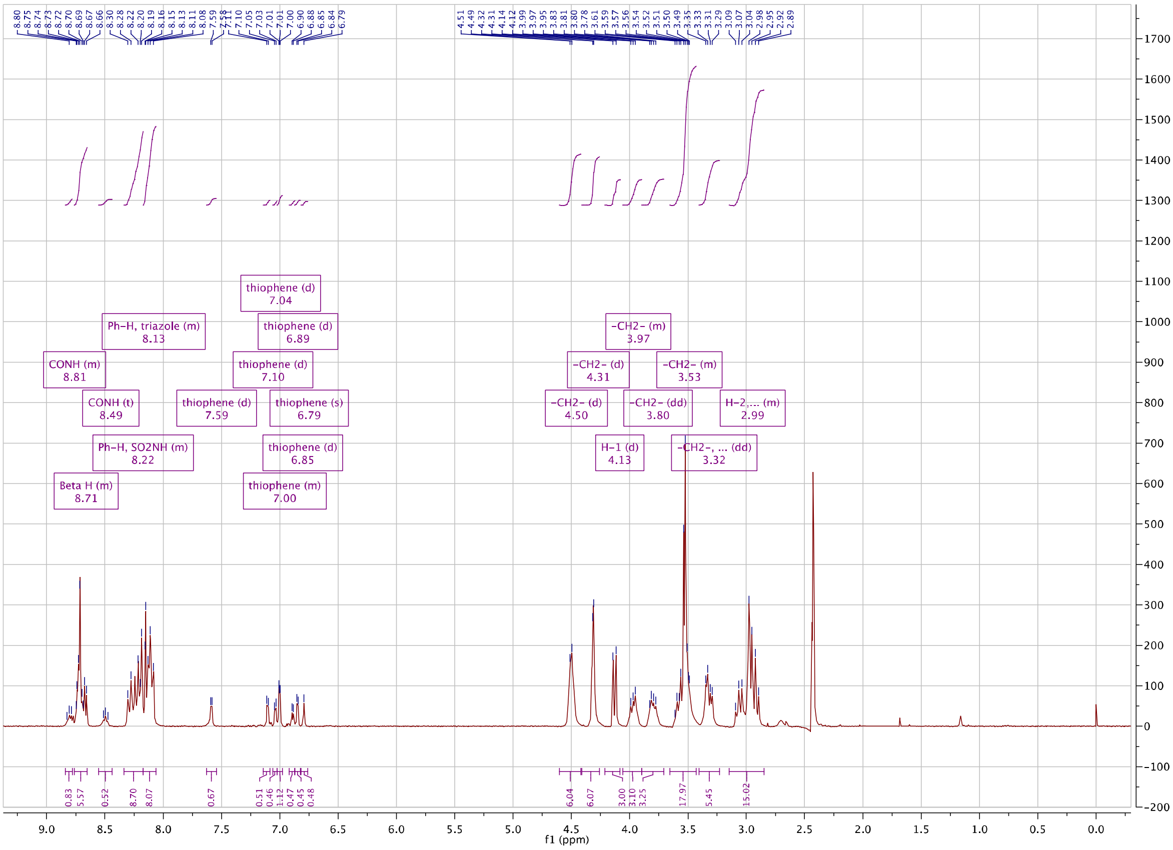

Ph−H, triazole (m) 8.13
CONH (m) 8.81
CONH (t) 8.49
Ph−H, SO2NH (m) 8.22
Beta H (m) 8.71
thiophene (d) 7.04
thiophene (d) 6.89
thiophene (d) 7.10
thiophene (d) 7.59
thiophene (s) 6.79
thiophene (d) 6.85
thiophene (m) 7.00
−CH2− (m) 3.97
−CH2− (d) 4.31
−CH2− (m) 3.53
−CH2− (d) 4.50
−CH2− (dd) 3.80
H−2,... (m) 2.99
H−1 (d) 4.13
−CH2−, ... (dd) 3.32
f1 (ppm)

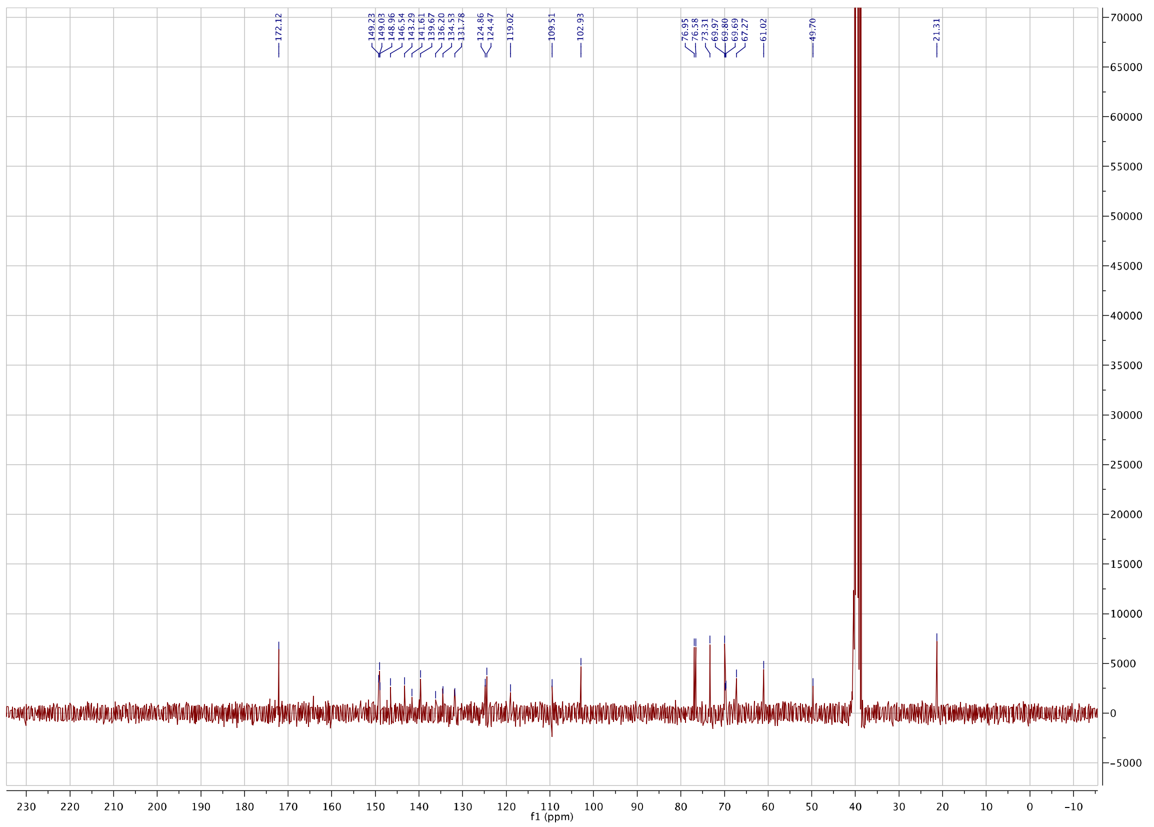

172.12
149.23
149.03
148.96
146.54
143.29
141.61
139.67
136.20
134.53
131.78
124.86
124.47
119.02
109.51
102.93
76.95
76.58
73.31
69.97
69.80
69.69
67.27
61.02
49.70
21.31
f1 (ppm)

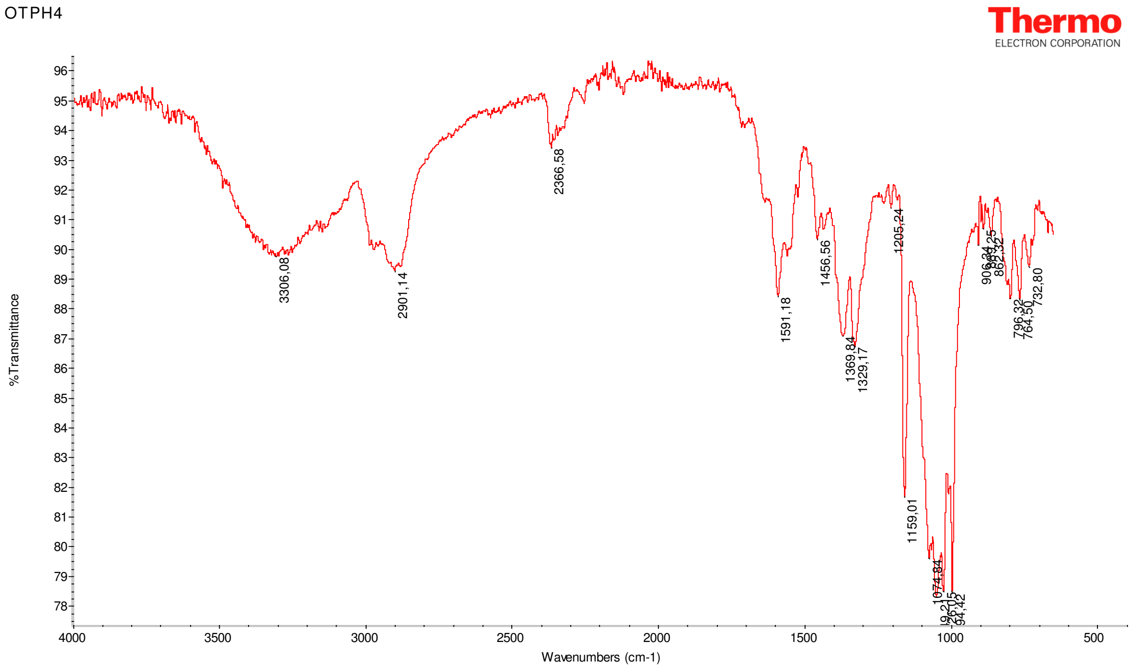

OTPH4
Thermo
ELECTRON CORPORATION
%Transmittance
Wavenumbers (cm-1)
3306,08
2901,14
2366,58
1591,18
1456,56
1369,84
1329,17
1205,24
1159,01
1074,84
796,32
764,50
732,80
Wed Apr 11 16:14:11 2018 (GMT+02:00)
